# The Effect of Life History on Retroviral Genome Invasions

**DOI:** 10.1371/journal.pone.0117442

**Published:** 2015-02-18

**Authors:** Ravinder K. Kanda, Tim Coulson

**Affiliations:** Department of Zoology, University of Oxford, South Parks Road, Oxford, United Kingdom; University of Athens, Medical School, GREECE

## Abstract

Endogenous retroviruses (ERV), or the remnants of past retroviral infections that are no longer active, are found in the genomes of most vertebrates, typically constituting approximately 10% of the genome. In some vertebrates, particularly in shorter-lived species like rodents, it is not unusual to find active endogenous retroviruses. In longer-lived species, including humans where substantial effort has been invested in searching for active ERVs, it is unusual to find them; to date none have been found in humans. Presumably the chance of detecting an active ERV infection is a function of the length of an ERV epidemic. Intuitively, given that ERVs or signatures of past ERV infections are passed from parents to offspring, we might expect to detect more active ERVs in species with longer generation times, as it should take more years for an infection to run its course in longer than in shorter lived species. This means the observation of more active ERV infections in shorter compared to longer-lived species is paradoxical. We explore this paradox using a modeling approach to investigate factors that influence ERV epidemic length. Our simple epidemiological model may explain why we find evidence of active ERV infections in shorter rather than longer-lived species.

## Introduction

A significant proportion of host genomes are littered with the remnants of past retroviral infections. Termed endogenous retroviruses (ERVs), in humans ERVs represent 8% of the genome and over 10% of the genome in mice [1,2]. Infection by a retrovirus requires integration into the cellular DNA as part of its replication cycle. Integration into the germline cells and subsequent vertical transmission provides us with a genomic fossil record of multiple, independent, ancient retroviral infections, described from a wide range of vertebrate genomes, including mammals, fish, birds, reptiles and amphibians [3,4]. Typically, an ERV consists of 3 genes (*gag*, *pol* and *env*) and two flanking non-coding long terminal repeats (LTRs), which are identical at the time of integration. Over time, these retroviral insertions accumulate mutations and deletions at the same rate as the mutation rate of the host genome [5], rendering them non-functional. ERVs may also be inactivated by recombinational deletion between the two flanking LTRs, which removes the internal coding region, leaving a solo LTR. Solo LTRs are 10–100 times more numerous than their full length counterparts [6], and many of these insertions are fixed in the host population. In various mammal species there are a few examples of intact, evolutionarily young ERVs that are polymorphic (present in some but not all individuals) in their host population [7–12]; whether this is persisting polymorphism maintained through various evolutionary forces or actual active ERVs remains to be established. However, some ERVs do appear to be intact, capable of expression and replication [11,13–17]—what we consider to be “active”.

To date, no active ERVs have been discovered in humans. Most ERV research in humans is computationally based, comprising of data mining of sequenced human genomes, which has revealed numerous polymorphic ERV insertions of the youngest known family of human ERVs, HERV-K(HML2) [7,18]. The most common insertions (those insertions present at high frequency in the population) are present in the reference genome; it is unclear whether the newer insertions more recently identified are evidence of activity of this particular retroviral family, or lingering polymorphism. However, until a particular insertion in the human genome reaches a high frequency, it is unlikely to be detected unless one is specifically looking for them [7]. By the same token, any new retroviral invasions into the human genome would also be unlikely to be detected until they reached an appreciable frequency in the population. In contrast, the other mammalian genome that has been extensively studied is that of the mouse. Their use as a model organism has led to a great deal of experimental research on mice, informing our understanding of the various roles that ERVs may play and revealing a number of active ERVs [2,12,16] (“active” in the sense that there are intact copies in the genomes which are capable of expression and replication—in some cases have been shown to produce infectious viral particles). This has led to the conclusion that mice have more frequent ERV invasions than humans [19], but is this true? To ascertain the activity of ERVs in different species would require the same amount of effort that has been invested in research on mice ERVs, which in most cases is unfeasible. A search through the literature shows that despite over 3500 endogenous retroviruses having been sequenced from 138 species of mammals, the majority of ERV research has focused on just a handful of species (Table 1 —see Methods for further details). An alternative approach is to model ERV dynamics, and explore factors that would influence our chance of detecting active ERVs, such as epidemic length (i.e. how long does it take for an endogenous retroviral insertion to reach a high enough frequency that it would be detected in the few whole genomes from a species that are sequenced?).

**Table 1:** Focus of ERV research across species (105) with different life histories.

Latin Name	Common Name	Total no. of ERVs identified	GestationLength	Effort	Active ERVs described
Ornithorhynchus anatinus	Platypus	1	12.48	0	0
Monodelphis domestica	Gray short-tailed opossum	1	14.55	1	0
Trichosurus vulpecula	Common brushtail	3	17.5	0	0
Mus cervicolor	Fawn-coloured mouse	1	17.6	3	0
Mus caroli	Ryukyu mouse	1	18.1	4	0
Mus musculus	Mouse	234	19.6	708	9
Myodes glareolus	Bank vole	4	19.74	0	0
Chionomys nivalis	European snow vole	3	20.92	0	0
Phodopus sungorus	Siberian hamster	5	21	1	0
Rattus norvegicus	Norway rat	10	21.74	2	1
Oryzomys palustris	Marsh rice rat	16	24.74	1	0
Peromyscus maniculatus	North American deer mouse	23	26.68	1	0
Sigmodon hispidus	Hispid cotton rat	11	27	1	0
Macropus eugenii	Tammar wallaby	4	29.89	3	0
Macropus rufogriseus	Red-necked wallaby	6	30	1	0
Suncus murinus	House shrew	4	30.19	2	0
Oryctolagus cuniculus	Rabbit	9	30.45	43	0
Phascolarctos cinereus	Koala	1	34.29	12	1
Microcebus murinus	Gray mouse lemur	4	60.34	3	0
Cheirogaleus medius	Fat-tailed dwarf lemur	3	61.79	0	0
Felis chaus	Jungle cat	3	62.88	1	0
Felis catus	Domestic cat	38	62.99	53	1
Canis lupus familiaris	Dog	5	63.5	16	0
Felis silvestris	Wildcat	4	65.49	2	0
Cavia porcellus	Guinea pig	5	66.99	10	0
Prionailurus bengalensis euptilurus	Leopard cat	1	70.18	0	0
Puma concolor	Puma	2	92.3	0	0
Dasyprocta leporina	Brazilian agouti	1	106.39	0	0
Chinchilla lanigera	Long-tailed chinchilla	1	112.47	0	0
Sus barbatus barbatus	Bearded pig	27	114.63	0	0
Sus barbatus oi	Bearded pig	26	114.63	0	0
Sus scrofa	Pig	441	115.2	281	1
Potamochoerus porcus	Red river hog	32	120	1	0
Eulemur fulvus	Brown lemur	1	120.83	0	0
Sus verrucosus	Java warty pig	27	121.66	0	0
Potamochoerus larvatus	Bush pig	29	121.73	1	0
Sus celebensis	Celebes wild boar	24	126	0	0
Aotus trivirgatus	Northern owl monkey	11	133.47	1	0
Lemur catta	Ring-tailed lemur	2	134.74	1	0
Ailuropoda melanoleuca	Giant panda	1	134.99	1	0
Saguinus midas	Red-handed tamarin	8	138.24	0	0
Callithrix jacchus	Marmoset	7	144	6	0
Cercopithecus ascanius	Red-tailed monkey	1	148.5	0	0
Hydrochoerus hydrochaeris	Capybara	1	150.73	0	0
Hylochoerus meinertzhageni	Giant forest hog	9	151	0	0
Ovis aries	Sheep	18	152.54	91	1
Babyrousa babyrussa	Babirusa	11	156.5	0	0
Saimiri boliviensis boliviensis	Bolivian squirrel monkey	2	157.79	0	0
Macaca cyclopis	Formosan rock macaque	2	161.06	0	0
Cebus capucinus	White-headed capucin	2	161.06	0	0
Pithecia pithecia	White-faced saki	2	161.13	0	0
Macaca radiata	Bonnet macaque	2	161.56	2	0
Saimiri sciureus	Squirrel monkey	9	164.09	23	0
Miopithecus talapoin	Angolan talapoin	3	164.38	1	0
Macaca fascicularis	Crab eating macaque	42	164.69	17	0
Macaca sylvanus	Barbary macaque	2	164.84	0	0
Cercocebus atys	Sooty mangabey	1	165.08	1	0
Phacochoerus aethiopicus	Desert warthog	18	165.4	0	0
Macaca mulatta	Rhesus macaque/monkey	27	166.07	61	0
Saguinus oedipus	Common-top tamarin	3	166.49	1	0
Macaca maura	Moor macaque	1	167.19	0	0
Erythrocebus patas	Patas monkey	1	167.2	0	0
Macaca thibetana	Tibetan macaque	2	169.02	0	0
Cercopithecus pogonias	Crested mona monkey	1	169.51	0	0
Cercopithecus nictitans	Greater spot-nosed monkey	1	169.51	0	0
Macaca nemestrina	Pig-tailed macaque	7	171	6	0
Macaca silenus	Lion-tailed macaque	2	172	1	0
Cercopithecus neglectus	De Brazza's monkey	2	172.07	0	0
Macaca nigra	Celebes crested macaque	1	172.43	0	0
Phacochoerus africanus	Warthog	33	172.49	1	0
Macaca fuscata	Japanese macaque	65	172.99	3	0
Papio cynocephalus	Yellow baboon	2	172.99	5	0
Mandrillus sphinx	Mandrill	2	173.99	1	0
Cercocebus galeritus	Tana River mangabey	1	174.43	0	0
Macaca arctoides	Stump-tailed macaque	4	176.6	3	0
Theropithecus gelada	Gelada baboon	2	178.64	0	0
Papio anubis	Olive baboon	8	178.96	2	0
Papio hamadryas	Hamadryas baboon	15	180	2	0
Macaca sinica	Toque macaque	2	180.9	0	0
Papio papio	Guinea baboon	2	184.42	1	0
Papio ursinus	Chacma baboon	3	185.92	0	0
Alouatta seniculus	Venezuelan red howler	7	189.9	0	0
Semnopithecus entellus	Hanuman langur	2	197.7	3	0
Hylobates pileatus	Pileated gibbon	2	200.16	0	0
Odocoileus hemionus	Mule deer	1	203.49	1	1
Hylobates lar	Gibbon	4	212.91	55	0
Symphalangus syndactylus	Siamang	2	230.66	1	0
Procavia capensis	Cape rock hyrax	20	231.24	0	0
Pan troglodytes	Chimpanzee	93	231.49	63	0
Pan paniscus	Bonobo chimp	25	235.24	2	0
Tragelaphus spekii	Sitatunga	1	241.15	0	0
Hylobates moloch	Silvery gibbon	2	241.2	0	0
Gorilla gorilla	Gorilla	56	257	32	0
Pongo pygmaeus	Orangutan	46	259.42	25	0
Homo sapiens	Human	1580	274.78	2082	0
Bos taurus	Cattle	11	280.48	44	0
Bos javanicus	Banteng	1	296.78	0	0
Bubalus bubalis	Water buffalo	1	320	0	0
Trichechus manatus	West Indian manatee	20	334.58	1	0
Equus caballus	Horse	7	338.97	7	0
Equus asinus asinus	Donkey	4	367	0	0
Globicephala macrorhynchus	Short-finned pilot whale	1	452	0	0
Orcinus orca	Killer whale	1	456.25	2	0
Elephas maximus	Asiatic elephant	41	634.49	0	0
Loxodonta africana	African elephant	42	660	0	0

Total number of ERVs identified represents the number of entries in the NCBI nucleotide database for each species. Species where information was not available in PanTHERIA were excluded for the purposes of this table. Gestation length is given in days. Effort represents the number of entries found in PubMed. Search criteria are described in the methods. Searches are correct as of 30/09/13.

Intuitively, there are two factors that affect the likelihood of finding active ERVs: the rate of incorporation/loss—if it varies with life history, and how long they take to run their course (i.e. the time to fixation at a specific locus), which we call the length of the epidemic. Little is known about how the rate of incorporation/loss may vary across species with different life histories. Here we explore how we would expect the length of an epidemic to vary across different life histories: where should we be looking to identify active ERVs? Given that organisms with a faster life history are i) short lived (shorter generation times), ii) tend to have larger numbers of offspring, and iii) have a larger effective population size, we might at first expect that a beneficial insertion (those ERVs that are involved in endogenous viral element derived immunity (EDI), and would therefore be considered beneficial) would spread to fixation faster in these species, than those with a slower life history [20]. Hence, we would expect an ERV epidemic to take longer (in years) in species with slower life histories and be more easily detectable at a given time. We explore how a search across different life histories may affect what we find.

Susceptible-Infectious-Recovered (SIR) models are a useful tool for inferring the length of an epidemic [21]. They have been used extensively to describe the dynamics of various infectious disease epidemics, including foot and mouth disease [22–24] and measles [25–27]. We have previously described a SIR model to investigate the circumstances under which a disease-causing retrovirus can become incorporated into the host genome and spread through the host population, if it confers an immunological advantage [28]. This use of compartmental models is now being used by others to investigate retroviral dynamics [29]. We use the model of Kanda *et al* [28] to explore which factors would influence the length of an epidemic of such an ERV (i.e. one involved in EDI), and how this varies with the life history of the host.

## Methods

A keyword search on the NCBI nucleotide database (http://www.ncbi.nlm.nih.gov/nuccore) for “endogenous retrovirus” AND “mammals” shows the number of endogenous retroviruses that have been described in mammals (3542 in 138 species—see S1 Table for accession numbers). Using PanTHERIA [30], we retrieved information on gestation length for 105 of these species. Species where information was not available in PanTHERIA, were excluded from this analysis. Gestation length has been shown to be a suitable indicator of speed of life history [31]. To assess the amount of “effort” focused on the ERVs of these species, we conducted a PubMed search using the “latin name” OR “common name” AND “endogenous retrovirus” as search terms (see S2 Table for PubMed search results). The number in the column headed “effort” represents the number of papers returned by PubMed. From the literature, we identified those species described as having active ERVs. This data is collated in Table 1 where species are ordered from faster life history to slow. From this table we examined the association between life history, measured as gestation length, and the number of active endogenous retroviruses reported having corrected for effort (measured as the number of papers on ERVs in the host species), using a generalized linear model (GLM) with Poisson distribution. This analysis, although relatively crude, will highlight evidence for any association between host life history and the number of active ERVs.

### The Model

Kanda *et al* [28] developed a set of epidemiological models to investigate the conditions under which incorporation of retroviruses into the host genome benefits the host. Here, we use one of these models to examine the effects of life history on epidemic length. This is a type of compartmental model, standardly used to study the dynamics of a range of diseases [32,33].

**Fig 1:**
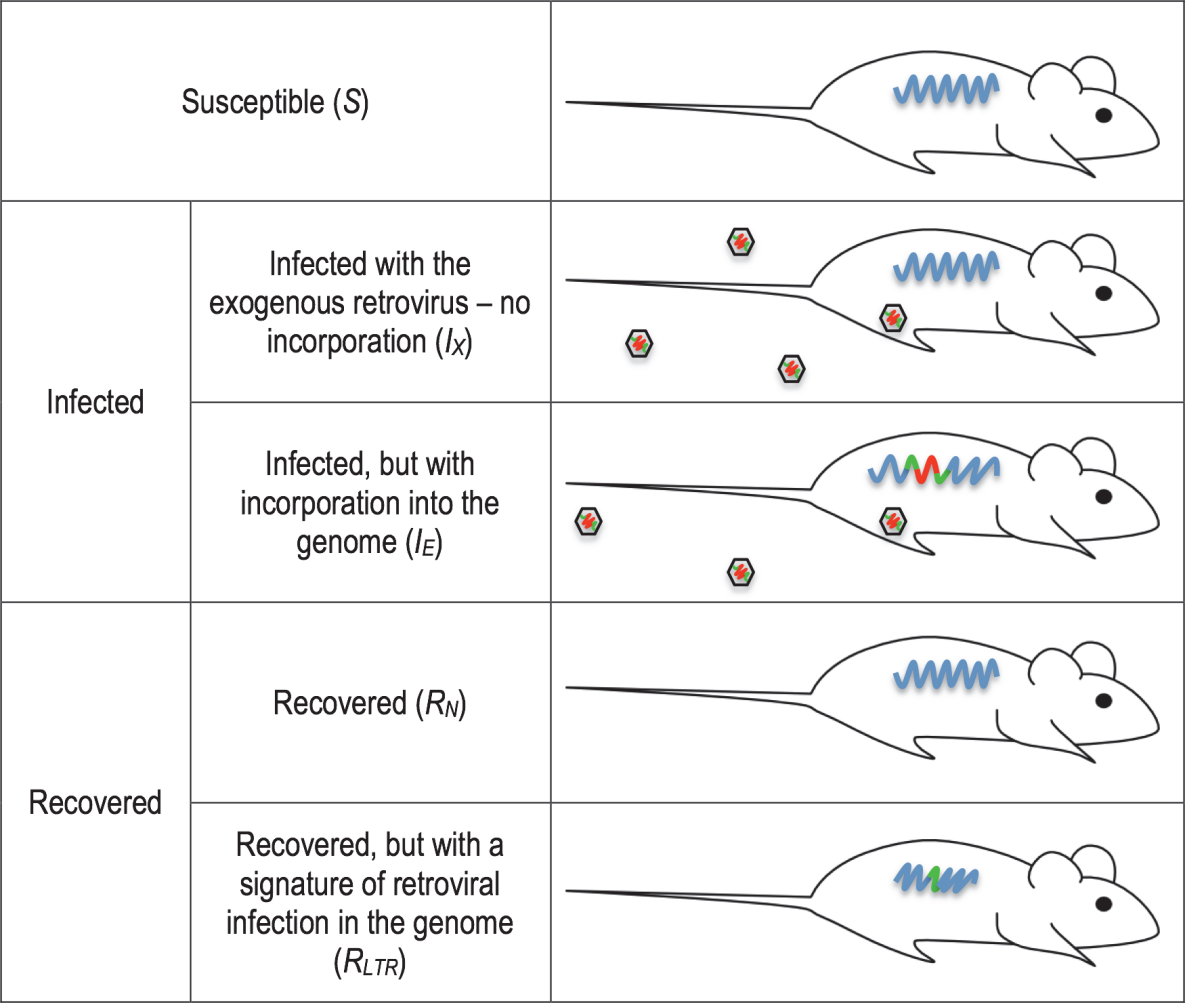
Overview of the different compartments in the SIR model. Susceptible individuals (S), have not encountered the retroviral infection. Those individuals in the I_X_ compartment have encountered exogenous viral particles and are infected, but the virus has not integrated into their genome. Integration of the retrovirus into the germline of these I_X_ individuals results in the offspring of these individuals entering the I_E_ compartment—these individuals are infected and infectious, and have an endogenous copy of the retrovirus in their genome. Individuals in the R_N_ compartment have successfully dealt with the infection, without incorporating the virus into their genome—they are the same as the S individuals. Individuals in the R_LTR_ compartment have also successfully dealt with the infection, but they are left with a copy of the integration in their genome (illustrated here as the LTR (green), but may also be a full length provirus (red and green as in the virus)).

**Fig 2:**
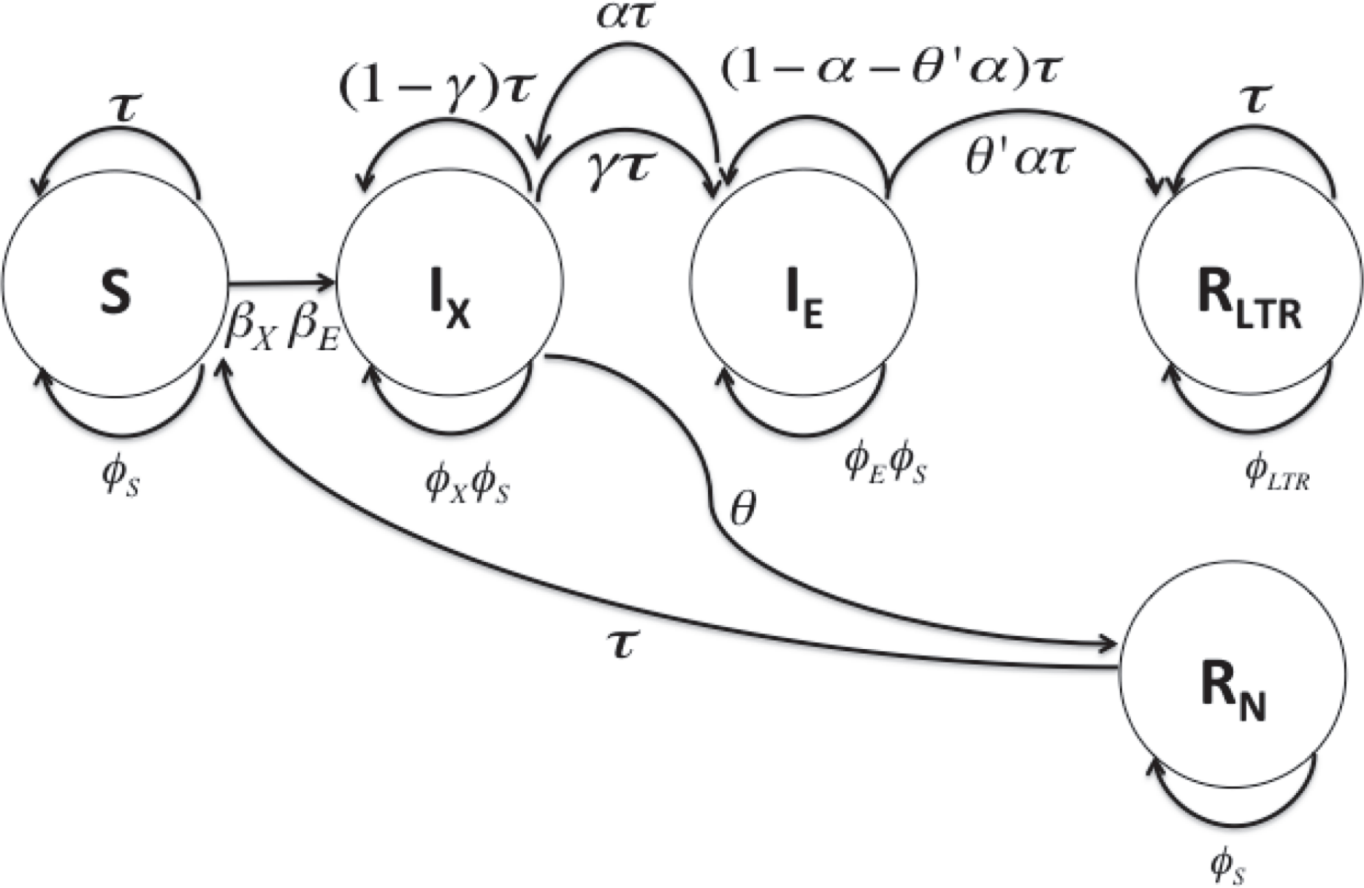
Graphical representation of the *SI*_*X*_ *I*_*E*_ *R*_*N*_ *R*_*LTR*_ model. The circles represent compartments; the arrows represent transition rates between the compartments. Upon infection with a retrovirus, susceptible individuals (S compartment) transition to the I_X_ compartment. If immunity is easily acquired to the retroviral infection, individuals enter the R_N_ compartment; they have recovered without integration of the retrovirus, and the offspring of these individuals enter the susceptible compartment. If it is difficult for immunity to arise to the retrovirus, then integration of the retrovirus into the germline of these I_X_ individuals may occur, and the offspring of these individuals enter the I_E_ compartment. After the threat of the retroviral infection has passed, the integration is free to be lost to recombinational deletion (or mutation), and individuals enter the R_LTR_ compartment.

We focus on the final model presented by Kanda *et al* [28] that realistically represented the dynamics of an EDI type ERV across a range of life histories. This model differs from a standard SIR model, in that it includes 2 infected compartments, *I*_*X*_ and *I*_*E*_ (Fig. 1). The *I*_*X*_ compartment refers to individuals who are infected with the exogenous retrovirus; upon successful incorporation of the retrovirus into the germline of an individual, the offspring of these individuals enter the *I*_*E*_ compartment—these individuals have an endogenous copy of the retrovirus. The model also has 2 recovered compartments, *R*_*N*_ and *R*_*LTR*_. Individuals in the *R*_*N*_ compartment have successfully dealt with the exogenous retroviral infection without incorporation of the retrovirus. The *R*_*LTR*_ compartment consists of individuals who have recovered from the retroviral infection with an endogenous copy of the retrovirus in the genome. The model is described mathematically by the equations below and represented graphically in Fig. 2. The equations describe the flow of individuals between compartments:

$$S\left(t+1\right)\;=\;\left(\phi_{s}+\tau \right)S\left(t\right)-\frac{\beta_{X}S\left(t\right)I_{X}\left(t\right)}{N\left(t\right)}-\frac{\beta_{E}S\left(t\right)I_{E}\left(t\right)}{N\left(t\right)}+\tau R_{N}\left(t\right)$$

$$I_{X}\left(t+1\right)\;=\;\left(\phi_{X}\phi_{S}+\tau \right)I_{X}\left(t\right)+\frac{\beta_{X}S\left(t\right)I_{X}\left(t\right)}{N\left(t\right)}+$$

$$+\frac{\beta_{E}S\left(t\right)I_{E}\left(t\right)}{N(t)}-\theta I_{X}\left(t\right)-\;\gamma \tau I_{X}\left(t\right)+\alpha {\tau I}_{E}\left(t\right)$$

$$I_{E}\left(t+1\right)\;=\;\left(\phi_{E}\phi_{S}+\tau \right)I_{E}\left(t\right)+\gamma \tau I_{X}\left(t\right)-\alpha {\tau I}_{E}\left(t\right)-\alpha {\tau \theta 'I}_{E}\left(t\right)$$

$$R_{N}\left(t+1\right)\;=\;\phi_{S}R_{N}\left(t\right)+\theta I_{X}\left(t\right)$$

$$R_{LTR}\left(t+1\right)\;=\;\left(\phi_{LTR}+\tau \right)R_{LTR}\left(t\right)+\theta '\alpha \tau I_{E}\left(t\right)$$ (1)

where *t* represents time, *τ* is the birth rate (which does not differ between compartments) and *ϕ_s_* is the survival rate of susceptible individuals. *γ* is the rate of incorporation of the retrovirus, *α* is the rate of loss of the endogenous retrovirus (mutation or recombinational deletion), *β_X_* and *β_E_* are the infection rates of the exogenous and endogenous virus respectively, *θ* and *θ*' are the rates at which immunity is acquired to the exogenous and endogenous virus respectively, *ϕ_X_ϕ_S_* and *ϕ_E_ϕ_S_* are the survival rates of the individuals infected by the exogenous and endogenous virus respectively. *N(t) = S(t) + I_X_(t) + I_E_(t) + R_N_(t) + R_LTR_(t)*, is the total population size. Baseline values and a description of the parameters are summarised in Table 2.

**Table 2:** Parameters and definitions in the SIR model.

Parameter	Baseline Values	Definition
ϕ_s_	1.016-τ	Survival rate per time step of an individual uninfected with a virus
ϕ_X_	0.97	The proportional reduction in ϕ_s_ caused by the exogenous retrovirus
ϕ_E_	0.985	The proportional reduction in ϕ_s_ caused by the endogenous retrovirus
τ	1.016-ϕ_s_	The birth rate per time step
β_X_	0.5	The infection rate of an individual with the exogenous virus
β_E_	0.4	The infection rate of an individual with the endogenous virus
γ	0.0001	Rate of incorporation
α	0.00001	Rate of loss of the incorporated virus
θ	0.05	The rate at which immunity is acquired to the exogenous retrovirus
θ'	0.01	The rate at which immunity is acquired to the endogenous retrovirus

Summary of the parameters in the model and their baseline values.

### Parameter values for the model

The survival (*ϕ_s_*) and fertility (τ) rate parameters define the life history of a species in this model [34]. Large *ϕ_s_* and small τ correspond to a species with a slow life history, while large τ and small *ϕ_s_* correspond to a species with a faster life history. We constrain *ϕ_s_* + τ = 1.016, as smaller values increase the probability of extinction, but above this value the extinction risk is virtually zero. We alter the life history of the species by changing the values of *ϕ_s_* and τ such that their sum always equals 1.016. As τ gets larger the life history speeds up, and as *ϕ_s_* increases the life history slows down. We vary *ϕ_s_* and τ in increments of 0.01. For each life history we then independently vary values of all other parameters as described below.

We model the invasion of an immunologically beneficial ERV, by imposing a conservative mortality increase of 1 - *ϕ_X_* = 0.03 on survival (new survival rate = *ϕ_X_ϕ_S_*) attributable to the exogenous virus. For the endogenous virus, we impose a mortality increase on survival of 1 - *ϕ_E_* = 0.015 (new survival rate = *ϕ_E_ϕ_S_*). *ϕ_E_* and *ϕ_X_* are reductions in *ϕ_S_* (survival). We assume that the mortality rate of the *I*_*E*_ group is less than that of the *I*_*X*_ compartment. Similarly, we assume the infection rates of the exogenous virus (*β_X_*) and the endogenous virus (*β_E_*) are also likely to differ, with the endogenous virus being less infectious than the exogenous. Baseline values are set at 0.5 and 0.4 respectively. We explore how varying these values (*β_X_* and *β_E_*) in increments of 0.05 affect the epidemic length across different life histories.

With the exception of a few HERVs, there is little information available regarding the rate of incorporation of the retrovirus (*γ*) and the rate of loss (*α*) of the virus (mutation or recombinational deletion). The estimates that are available for humans suggest that *α*<*γ* (see [28] for further details). Our baseline values are set at *α* = 0.00001 and *γ* = 0.0001 We explore how varying these two parameters affects the length of the epidemic (from 0.0001 to 0.001).

The rates at which immunity would arise to the exogenous virus (*θ*) and the endogenous virus (*θ*') are unknown and so we set the baseline values for these parameters at *θ* = 0.05 and *θ*' = 0.01; we have previously shown the absolute parameter values of *θ*, *θ'* and *α* are relatively unimportant, as it is their relative values that determine the dynamics [28]. As the values of *θ*, *θ'* and α approach zero, the longer the epidemic lasts and the longer simulations need to be run until the asymptotic equilibrium is reached. We vary *θ* and *θ*' from 0.0001 to 0.1 in 10 increments to determine the influence of immunity on the length of an epidemic.

### Conducting the simulation

We ran simulations of the model until the proportion of the population in each class was stable to a tolerance of 0.000001, and recorded the length of time until equilibrium. We start with one *I*_*X*_ individual and the rest of the population in the *S* compartment. The simulation was run for a maximum of 50000 iterations to allow the population to reach a stable equilibrium, and we calculated the number of generations this had taken. Generation length (*T_C_*) was calculated using the equation below [35]:

$$T_{C}\;=\;\frac{\Sigma al_{a}m_{a}}{\Sigma l_{a}m_{a}}\;=\;\frac{\Sigma a{\phi_{s}}^{a}\tau }{\Sigma {\phi_{s}}^{a}\tau }$$ (2)

where *a* represents age, *l_a_* (or *ϕ* *_s_^a^*) is the survivorship from birth to age *a*, and *m_a_* (or *τ*) is the fertility rate at age *a*. Baseline values for the various parameters are described above. All simulations were conducted in *R* version 2.15.2 [36].

### Sensitivity of epidemic length to transition rates

Because we are interested in the effect of the range of parameter values on the length of the epidemic across life histories, we wish to examine how varying the model parameters impacts the time taken for the population to reach equilibrium. We do this by systematically altering the values of each model parameter in 10 increments (between the ranges described for each parameter above), and re-running the simulation for different life histories (differing values of *ϕ*_*s*_ and τ).

## Results

**Fig 3:**
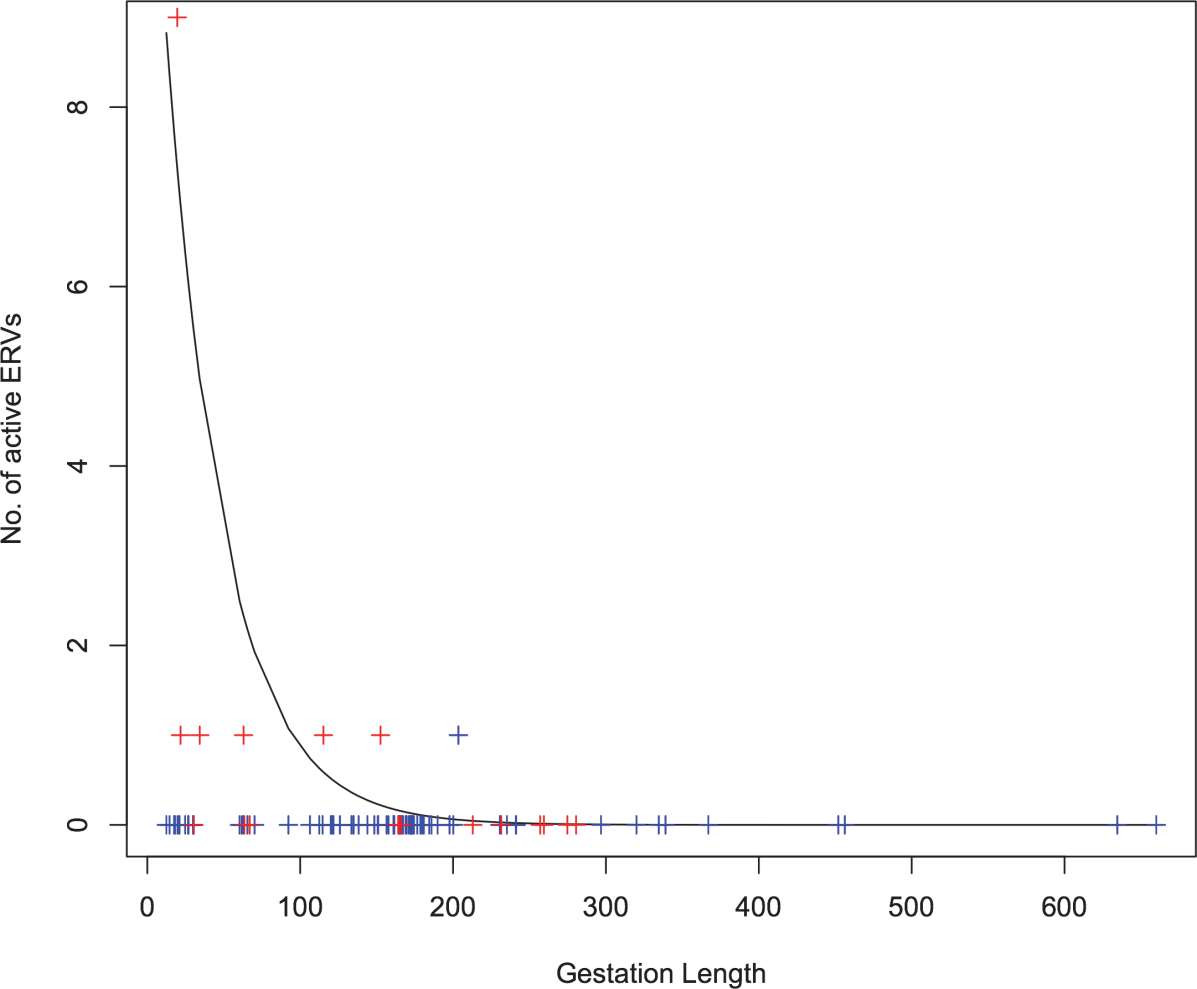
Predicted & observed number of active ERVs as a function of life history. Life history is approximated by gestation length (see Table 1). The black line represents the GLM predictions, whereas the crosses (blue and red) represent the actual data (from Table 1). Crosses in red indicate a high amount of “effort” (>10).

**Fig 4:**
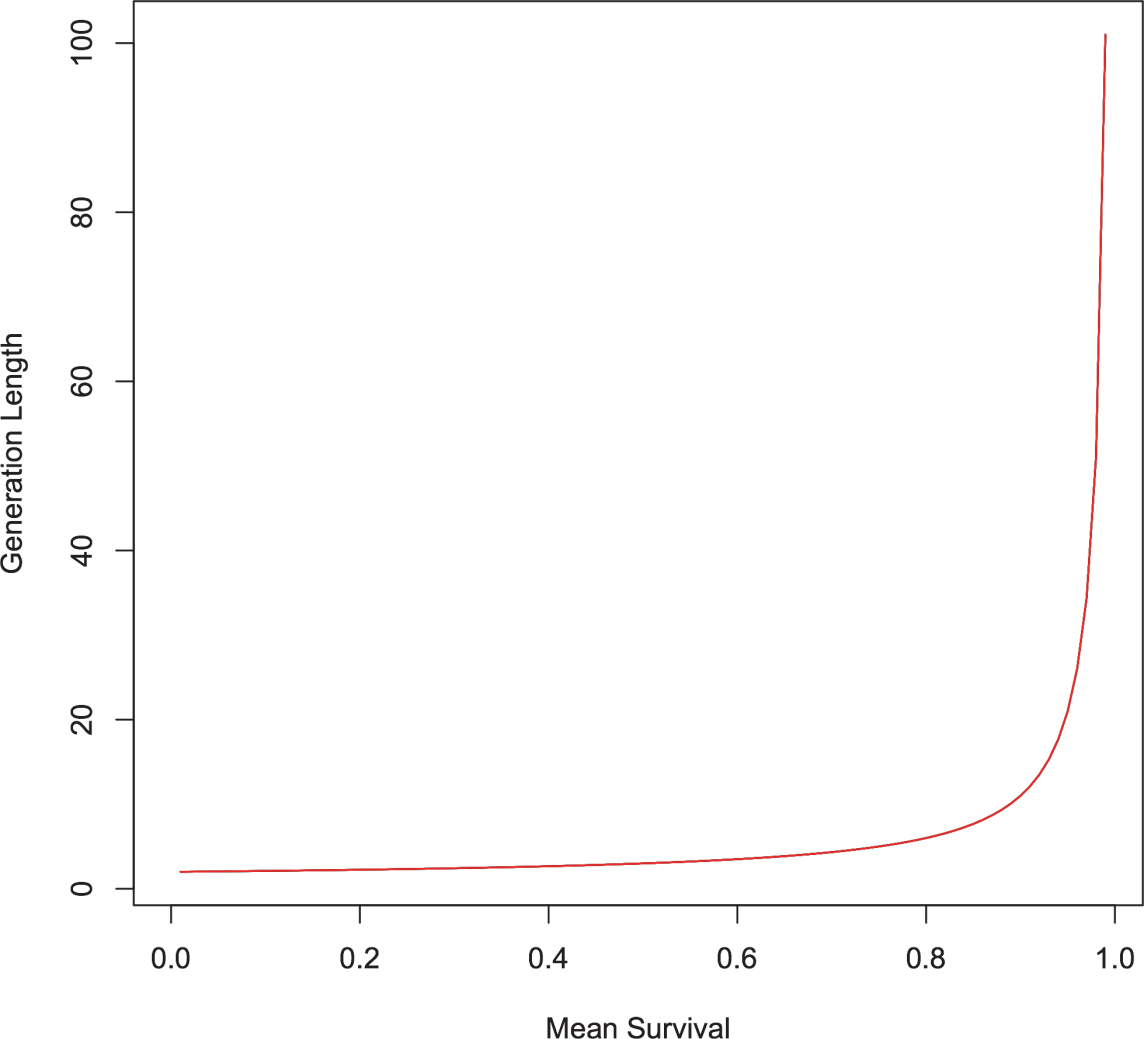
Relationship between generation length and mean survival. Given that mean survival and mean fertility are constrained (*ϕ_s_* + τ = 1.016), generation length increases as mean survival increases.

Analysis of data in Table 1 revealed a negative association between host gestation length and the number of active ERVs identified in the host species, corrected for effort (slope = -0.0264, s.d. = 0.0005, p < 0.001). Fig. 3 illustrates the effect of life history, as predicted by the GLM (line) compared to the actual data (crosses), on the number of active ERVs we see. The number of active ERVs significantly increases with the speed of host life history (as approximated by gestation length). In species where data is available, active ERVs are significantly more likely to be found in species with faster life history. This contrasts with our original intuitive expectation that ERV epidemics should run their course faster in fast-lived species, and that we should see less active ERVs in fast-lived species than slow-lived species. The relationship between the survival rate (*ϕ*_*s*_) and fertility rate (τ) is illustrated in Fig. 4. Given that mean survival and mean fertility are constrained, generation length increases as mean survival increases. Generation length, by its definition, is a function of *ϕ*_*s*_ and τ, and is unaffected by the other parameters (equation 2).

**Fig 5:**
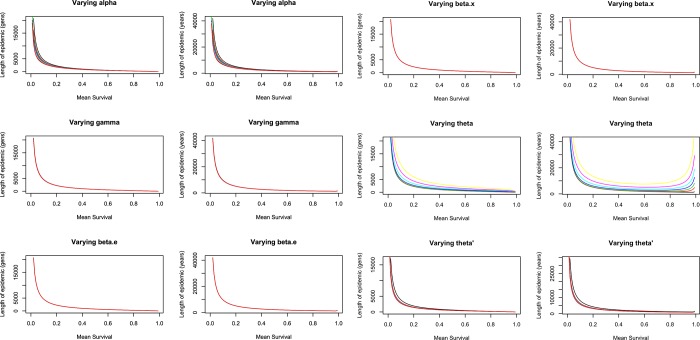
Effects of varying parameters on the length of the epidemic. Effect of varying the rate of loss (*α*) in a) generations and b) years, varying the rate of integration (*γ*) in c) generations and d) years, varying the rate of exogenous retroviral infection (*β_E_*) in e) generations and f) years, varying the rate of endogenous retroviral infection (*β_X_*) in g) generations and h) years, varying the rate at which immunity arises to the exogenous retrovirus (*θ*) in i) generations and j) years, and varying the rate at which immunity arises with the endogenous retrovirus (*θ*') in k) generations and l) years. The pattern is similar for most parameters, and is very pronounced, going from long epidemic lengths in short-lived species to short epidemic lengths in long-lived species. Parameter values are as described under “Methods—Parameter values for the model”.

The effects on the epidemic length, of varying the other transition parameters are illustrated in Fig. 5. The first striking observation overall, is that the length of the epidemic is more strongly correlated with generation length than anything else—something that is also suggested by data available from the literature (Fig. 3). The length of the epidemic is greater in species with a fast life history (short lived) than slow life histories, regardless of the parameters that describe infection rates, incorporation rate or rate of loss. For an epidemic to last, there must to be more individuals in the susceptible compartment, and in fast-lived species we have faster generation of susceptible individuals. Loss and integration can only occur during reproduction. As *τ* (birth rate) increases, the rate of flow of individuals into the I_X_ compartment increases much more than the rate of flow of individuals to the R_LTR_ compartment i.e. *α* > *θ'α* and it increases at a rate of $\frac{1}{\theta }$ – hence the epidemic takes longer to run its course in species with a fast life history. Secondly, we observe that when it is easy to acquire immunity to the exogenous retrovirus (higher values of *θ*), individuals spend more time moving around the left hand side of Fig. 2 (between the S, I_X_ and R_N_ compartments), and the epidemic lasts longer (Fig. 5 I and J). If immunity to the exogenous virus is easy to acquire, individuals never progress to the *I*_*E*_ compartment and the epidemic never progresses. This effect is less pronounced in species with longer life histories. At *θ* > 0.07, the population fails to reach a stable equilibrium in 50000 years [28].

The effect of most of the other parameters is negligible, with the exception of the rate of loss, *α* (Fig. 5 A and B), for species with a faster life history. As *α* increases, more individuals transition from the *I*_*E*_ compartment to the *I*_*X*_ compartment. This flow of individuals towards the left hand side of Fig. 2 results in the epidemic taking longer.

## Discussion

Our approach of using SIR models to study the multigenerational dynamics of an endogenous retroviral infection is a novel approach, combining methods from demography [35] with epidemiological methods to address the question of ERV epidemic length. Because our model is density independent, the population size will increase exponentially, but population growth rate and structure will converge on a stable equilibrium.

### Why are we interested in finding active ERVs?

There is a growing body of evidence to suggest that for some ERVs, incorporation into the genome provides the host with some immunity from related exogenous retroviruses (described as endogenous viral element derived immunity, EDI [37]), through a variety of different mechanisms [10,38–41]. Under this scenario, there is clearly an advantage to incorporation of an exogenous virus into the genome. However, despite the advantages of incorporation of retroviruses into the genome, there are also downsides to consider.

In many species there are numerous examples of ERVs causing disease [10,42,43]. In humans alone, HERVs have been associated with cancer [44–46], multiple sclerosis [47–49], and a whole host of other diseases (see [50] for review). Additionally, we also have to consider the possibility that the reverse of endogenisation may also occur—exogenous viruses emerging from active ERV lineages. The reservoir of viruses present in host genomes may also be able to recombine with exogenous retroviruses, resulting in novel recombinant viruses that maybe pathogenic—a phenomenon that has already been observed in cats [51]. It has been shown that the standard mechanism of ERV replication within a genome involves reinfection of germline cells, and hence possibly movement between host individuals [52,53]. Subsequently, cross-species transmission of these viruses is a very real concern—there are numerous examples identified to date, where cross-species transmission of retroviruses can lead to emergent disease, *e*.*g*. HIV was certainly acquired from the non-human primate version of the virus, SIV, which has crossed the species barrier on multiple occasions from chimpanzees and sooty mangabeys [54]—SIV has also been found to be endogenous in a species of lemur [39]. Other recent cross-species transmission event include the introduction of the koala retrovirus (KoRV), which is suspected to have originated from the exogenous Gibbon ape Leukemia Virus (GaLV) [55]. There are also several cases of close evolutionary relationships between exogenous retroviruses and ERVs in the same host species, *e*.*g*. in the sheep, cat, chicken and mouse [13,56]. Interpreting ERV diversity remains challenging and a better understanding of where we are likely to find active ERVs, and consequently possible threats of emerging disease, is clearly important in informing the direction of research in this area.

### Where and when would we expect to find active ERVs?

Our model suggests that the reason why more active ERVs are discovered in species with a fast life history (such as mice and koalas), than in those with a slow life history, is not necessarily that these species have more frequent ERV epidemics, but that those epidemics last longer and are therefore more likely to be detected. For the subset of ERVs involved in EDI, the life history of the host has the greatest bearing on the length of an epidemic. The next most influential factor is the rate at which immunity arises to the exogenous retroviral infection (*θ*). There is a greater rate of generation of susceptible individuals in faster life histories than slower, resulting in a longer time taken for the majority of the population to reach the R_*LTR*_ compartment. Not all ERVs will provide an immunity advantage. Our model applies to that subset of ERVs that are involved in EDI. The majority of active ERVs that have been described, are described in species that do have a faster life history, such as mice, koala and sheep [10,16,42], which would be in line with our predictions that these epidemics last longer in species with a fast life history, and are therefore more easily detectable. The unusually high number of active ERVs identified in mice may indeed be an anomaly; the numerous studies of ERVs in mice have focused on laboratory strains and it is possible that inbreeding and selection of certain characteristics of this model organism may have unintentionally contributed to the high levels of ERV activity observed in this particular species. For example, the first inbred mouse strain, DBA, was bred for its coat colour which has been shown to be the result of an ERV insertion [57]. However, until an equal amount of “effort” is invested into other species with similar life histories (or wild mice populations), it is difficult to ascertain whether mice are simply more susceptible to ERV infections. Nonetheless, more active ERVs are described in species with a fast life history. Table 1 illustrates the number of active ERVs that have been identified in all mammals in which ERVs have been described. There is a strong correlation between the number of active ERVs and the life history of the host (as estimated from gestation length), when weighted for effort, supporting our finding that we are more likely to find active ERVs in shorter lived species than in longer lived species (Fig. 3). Previously, these observations have been attributed to a higher level of activity of ERVs in these species (particularly in mice) [58–60]. In our model, this would correspond to higher values of infection rates (*β_X_* and *β_E_*), and incorporation (*γ*), which interestingly, do not appear to have a considerable effect on the length of an epidemic. In light of these results, it is also worth noting that the proportion of the genome derived from ERVs in mice (short life history) and humans (long life history) is fairly similar—8% and 10% respectively [1,2]. If ERV activity were greater in species with short life histories, then we should expect more of their genomes to originate from ERV insertions. The implications of these results are that in species with a slow life history (such as humans), we should not expect to easily find active ERVs, as the epidemic occurs quickly.

Existing data on species where active ERVs have been discovered are consistent with the results of our model. However, we acknowledge that this could also be a bias in the available data, as more research in this respect has been conducted on species with faster life histories, as demonstrated in Table 1. The model we have described does not account for specific mechanisms of EDI; a better understanding regarding the mechanisms behind how retroviral immunity (EDI) is gained, which may vary with life history, would be valuable in refining this model and allowing us to better target the search for active ERVs. Further studies of a range of species, with more active ERVs and contrasting life histories, will enable a better estimation of the parameters. However, our model suggests efforts to identify active ERVs should be focused on species with faster life histories, as this is where we stand a better chance of discovering active ERVs and potential threats of new emerging infections.

## Supporting Information

S1 Table Accession numbers for the 3542 endogenous retroviruses identified in the NCBI nucleotide database. (DOCX)

S2 Table Pubmed search results to assess the effort focused on the ERVs of a particular species. (XLSX)
