# Supplementary material for: The Effect of Life History on Retroviral Genome Invasions
Source: PLoS One. 2015 Feb 18;10(2):e0117442. doi: 10.1371/journal.pone.0117442 (PMC4333357; doi:10.1371/journal.pone.0117442)
Supplement: S1 Table — (DOCX) [file pone.0117442.s001.docx]

**Table S1 Accession numbers for the 3542 endogenous retroviruses identified in the NCBI nucleotide database**

AB025041.1

EU853148.1

EU853149.1

AY224323.1

AY224334.1

AY224335.1

AY224338.1

AY224339.1

AY224342.1

AY224343.1

AY224347.1

AY224350.1

EU853146.1

EU853147.1

NM_001190831.1

NM_001261719.1

AY224351.1

AY224354.1

AY224355.1

AY224367.1

AY224368.1

AY224362.1

AY224327.1

AY224365.1

AY224366.1

AF142988.1

AY224373.1

AY224375.1

AY224374.1

NM_001135588.1

EU853144.1

EU853145.1

NM_001080784.2

EU853150.1

EU853154.1

X57147.1

K02167.1

M11119.1

M85205.1

M12140.1

M12855.1

K03498.1

M14123.1

M12851.1

M12854.1

M12853.1

M12852.1

M12850.1

K03499.1

M74509.1

AB239248.1

AJ507119.1

AJ507120.1

AB025043.1

AB099389.1

AB099390.1

AB099391.1

AB099392.1

AB099393.1

AB099394.1

AB099395.1

EU853151.1

AF164897.1

AF164892.1

L38796.1

L38798.1

L38800.1

L38797.1

AF018153.1

AB025041.1

AB055744.1

AB055745.1

AB055746.1

AB055747.1

AB055748.1

DQ247961.1

EU853148.1

EU853149.1

HM347351.1

HM347352.1

KC010503.1

KC010515.1

AF315790.1

AF315791.1

AF315792.1

AF164886.1

AY224323.1

AY224324.1

AY224325.1

AY224326.1

AF018154.1

AF018155.1

AB055752.1

AB055753.1

AB055754.1

AB055755.1

AB055756.1

AB055757.1

AB055758.1

AB063622.1

AB063623.1

AB063624.1

AB087870.1

AB087871.1

AB087872.1

AB087873.1

AY224332.1

AY224333.1

AY224334.1

AY224335.1

AY224336.1

AY224337.1

AY224338.1

AY224339.1

AY224340.1

AY224341.1

AJ577599.1

AY425962.1

HM347355.1

HM347356.1

CM001293.1

JN985533.1

AB024481.1

AB024482.1

AB025039.1

AB071979.1

AB071980.1

AB071981.1

AB071982.1

AB073284.1

AB073285.1

AB073286.1

AB073287.1

AB073288.1

AB073289.1

AB073290.1

AB073291.1

AB073292.1

AB073293.1

AB087865.1

AB087866.1

AB087867.1

AB091010.1

AB091011.1

AB091012.1

AB091013.1

AB091014.1

AB091015.1

AB091016.1

AB091017.1

AB091018.1

AB091019.1

AB091020.1

AB091021.1

AB091022.1

AB091023.1

AB091024.1

AB091025.1

AB091026.1

AB091027.1

AB091028.1

AB091029.1

AB091030.1

AB091031.1

AB091032.1

AB091033.1

AB091034.1

AB091035.1

AB091036.1

AB091037.1

AB091038.1

AB091039.1

AB091040.1

AB091041.1

AY224342.1

AY224343.1

AY224344.1

AB198938.1

AB239199.1

AB239222.1

AB239223.1

AB239224.1

AB239225.1

AB239242.1

AB239243.1

AB239244.1

AB239245.1

L38802.1

AB025042.1

AF227991.1

AF227996.1

AF315793.1

AF315794.1

AB073294.1

AB073295.1

AB073296.1

AB073297.1

AB087868.1

AB087869.1

AY098484.1

AY224347.1

AY224348.1

AY224349.1

AY224350.1

DQ179114.1

NW_001106524.1

NC_007876.1

NR_003926.1

EU853146.1

EU853147.1

XM_002801387.1

XM_002801388.1

NM_001190831.1

NM_001261719.1

KC010501.1

KC010513.1

AY224351.1

AY224352.1

AY224353.1

AY224354.1

AY224355.1

AY224356.1

AY224357.1

DQ247962.1

AY224367.1

AY224368.1

AY224361.1

AY224362.1

AY224327.1

AY224328.1

AY224365.1

AY224366.1

AF164889.1

AJ507118.1

AY934762.1

DQ180329.1

HM347353.1

HM347354.1

XM_003897056.2

XM_003916061.2

AF142988.1

AF164887.1

AF164885.1

AB024483.1

AB024484.1

AB024485.1

AB024486.1

AB024487.1

AB024488.1

AB025040.1

AF315795.1

AF315796.1

AB055749.1

AB055750.1

AB055751.1

AY070776.1

AY224373.1

AF164894.1

AY224375.1

AF164890.1

AY224374.1

AB018370.1

AB018371.1

AB024473.1

AB024474.1

AB024475.1

AB024476.1

AB024477.1

AB024478.1

AB024479.1

AB024480.1

AB025038.1

AB087861.1

AB087862.1

AB087863.1

AB087864.1

AB198937.1

AB239198.1

AB239218.1

AB239219.1

AB239220.1

AB239221.1

AB239241.1

AY536064.1

DQ180331.1

KC010500.1

KC010512.1

AY934761.1

AY101592.1

AY101593.1

AF227992.1

AF227997.1

L38799.1

AF026253.1

AF026254.1

AF026255.1

AF094515.1

AB018366.1

AB018367.1

AF095799.1

AF095805.1

AF095811.1

AF165232.1

AF165244.1

AF165251.1

AF165256.1

AF165264.1

AB024462.1

AB024463.1

AB024464.1

AB024465.1

AB024466.1

AB025036.1

AF227994.1

AF227999.1

AJ291355.1

AJ291356.1

AJ291357.1

AJ291358.1

AJ291359.1

AJ507116.1

AJ507117.1

AJ507128.1

AB087856.1

AB087857.1

AB087858.1

AJ577596.1

AY101588.1

AY101589.1

AY425963.1

AY934760.1

AJ862655.1

AB198935.1

DQ180332.1

AB239194.1

AB239195.1

AB239210.1

AB239211.1

AB239212.1

AB239213.1

AB239214.1

AB239237.1

AB239238.1

KC010498.1

KC010510.1

NW_004006942.1

NC_018443.1

XM_004038670.1

XM_004038671.1

XM_004061361.1

AF095798.1

AF095804.1

AF095810.1

AF165231.1

AF165238.1

AF165239.1

AH008278.1

AF165243.1

AF165250.1

AF165255.1

AF165262.1

AF165263.1

AH008284.1

AB025034.1

AB239193.1

AB239207.1

AB239208.1

AB239209.1

AB239234.1

AB239235.1

AB239236.1

XM_003806455.1

XM_003806456.1

XM_003827553.1

K02915.1

L38806.1

AF026256.1

AF026257.1

AB018364.1

AB018365.1

AF139840.1

AF095797.1

AF095803.1

AF095809.1

AF165230.1

AF165236.1

AF165237.1

AH008277.1

AF165242.1

AF165249.1

AF165260.1

AF165261.1

AH008283.1

AB024457.1

AB024458.1

AB024459.1

AB024460.1

AB024461.1

AB025035.1

AF227995.1

AF228000.1

AJ291349.1

AJ291351.1

AJ291352.1

AJ291350.2

AJ291353.2

AF311937.1

AY098485.1

AJ507114.1

AJ507115.1

AJ507127.1

AB087855.1

AJ577595.1

AY101586.1

AY101587.1

AY934759.1

AJ862646.1

AJ862647.1

AJ862648.1

AJ862649.1

AJ862650.1

AJ862651.1

AB198934.1

DQ112112.1

DQ112125.1

DQ112126.1

DQ112128.1

DQ112129.1

DQ112130.1

DQ112133.1

DQ112135.1

DQ112136.1

DQ112137.1

DQ112138.1

DQ112141.1

DQ112142.1

DQ112144.1

DQ112145.1

DQ112149.1

DQ112150.1

DQ112153.1

DQ112154.1

DQ112155.1

DQ112156.1

DQ180330.1

DQ247959.1

AB239191.1

AB239192.1

AB239204.1

AB239205.1

AB239206.1

AB239231.1

AB239232.1

AB239233.1

NM_001135588.1

EU853144.1

EU853145.1

NM_001080784.2

GU459323.1

NW_003458524.1

NW_003457102.1

NC_006486.3

NC_006473.3

NW_003457182.1

NC_006474.3

KC010497.1

KC010509.1

XM_003310377.2

XM_001174645.4

XM_003316621.2

L38803.1

L38804.1

L38801.1

L38805.1

AB018368.1

AB018369.1

AF095800.1

AF095806.1

AF095812.1

AF165233.1

AF165245.1

AF165252.1

AF165257.1

AF165265.1

AB024467.1

AB024468.1

AB024469.1

AB024470.1

AB024471.1

AB024472.1

AB025037.1

AF227993.1

AF227998.1

AJ291354.1

AJ291360.1

AJ291361.1

AJ291362.1

AJ291363.1

AF375643.1

AJ507126.1

AB087859.1

AB087860.1

AJ577597.1

AY101590.1

AY101591.1

AJ862652.1

AJ862654.1

AB198936.1

AB239196.1

AB239197.1

AB239215.1

AB239216.1

AB239217.1

AB239239.1

AB239240.1

EU853150.1

EU853154.1

EU853155.1

NW_002890429.1

NC_012610.1

XM_002816423.1

KC010499.1

KC010511.1

XM_002814792.2

XM_002829690.3

Z12142.1

X06274.1

X06273.1

X06275.1

X06276.1

X06277.1

X06278.1

X06279.1

X07417.1

X07418.1

X07419.1

Z11739.1

Z11740.1

X57147.1

L09707.1

L09706.1

L09708.1

K02166.1

K02167.1

AH001453.1

M10976.1

J00273.1

J00274.1

J00275.1

M11119.1

M85205.1

K02016.1

K02269.1

K02270.1

K02271.1

K02017.1

AH002687.1

M12140.1

M12855.1

K03498.1

M14123.1

M12851.1

M12854.1

AH001458.1

M12853.1

M12852.1

AH001459.1

M12850.1

K03499.1

K02916.1

K02917.1

K02918.1

K02919.1

AH001460.1

M10935.1

K03431.1

D10083.1

D11078.1

S61070.1

M74509.1

M12211.1

M64936.1

M18048.1

M27828.1

M15971.1

X74434.1

U09236.1

S70570.1

S70571.1

S70572.1

S70573.1

M27826.1

X89211.1

X77055.1

X77054.1

X77056.1

X77058.1

X77062.1

X77060.1

X77059.1

X77061.1

X77068.1

X77063.1

X77065.1

X77071.1

X77057.1

X77064.1

X77070.1

X77066.1

X77069.1

X77067.1

X77072.1

X77073.1

U37251.1

U37263.1

U60268.1

U60269.1

U60270.1

U60271.1

U60272.1

U60273.1

U60274.1

S46006.1

U62098.1

U71456.1

U64453.1

U64455.1

D87055.1

D87056.1

D87057.1

D87058.1

AD000090.1

U88899.1

U88900.1

U88903.1

U88904.1

U88905.1

U88906.1

U88895.1

U88896.1

U87587.1

U87588.1

U87589.1

U87590.1

U87591.1

U87592.1

U87593.1

U87594.1

U87595.1

U87596.1

U07856.1

AF009361.1

AF015994.1

AF015995.1

AF015996.1

AF015997.1

AF015998.1

AF015999.1

AF016000.1

AF016001.1

AF026246.1

AF026247.1

AF026248.1

AF026249.1

AF026250.1

AF026251.1

AF026252.1

AF021928.1

AF021930.1

AC003973.1

AH005824.1

AC004761.1

AC004768.1

X16660.1

AC005179.1

AC005218.1

AC005217.1

AF020092.1

AF078838.1

AC005576.1

AC005574.1

AC005592.1

AF080618.1

AC005600.1

AF080229.1

AF080230.1

AF080231.1

AF080232.1

AF080233.1

AF080234.1

AC005723.1

AF058907.1

AC005946.1

AJ233628.1

AJ233629.1

AJ233630.1

AJ233631.1

AJ233632.1

AJ233673.1

U88901.1

U88902.1

AB016195.1

AB016525.1

AB016526.1

AB016527.1

AB016528.1

AF070683.1

AF070684.1

AF070685.1

AF070686.1

AF110315.1

AF064190.1

AF064191.1

AF072494.1

AF072495.1

AF072496.1

AF072497.1

AF072498.1

AF072499.1

AF072500.1

AF072501.1

AF072502.1

AF072503.1

AF072504.1

AF072505.1

AF072507.1

AF072508.1

AF069508.1

AF023339.1

AF109183.1

AF072506.2

AF079797.1

AF134984.1

AF108838.1

AF108841.1

AF108839.1

AF108842.1

AF108840.1

AF108843.1

AF087913.1

AF127226.1

NM_004929.2

AF159093.1

AF159094.1

AF159095.1

AF164609.1

AF164610.1

AF164611.1

AF164612.1

AF164613.1

AF164614.1

AF164615.1

AF095795.1

AF095796.1

AH008267.1

AF095801.1

AF095802.1

AH008268.1

AF095807.1

AF095808.1

AH008269.1

AF165228.1

AF165229.1

AH008275.1

AF165234.1

AF165235.1

AH008276.1

AF165240.1

AF165241.1

AH008279.1

AF165246.1

AF165247.1

AF165248.1

AH008280.1

AF165253.1

AF165254.1

AH008281.1

AF165258.1

AF165259.1

AH008282.1

AF097026.1

AF097027.1

AF179225.1

AF148679.1

AF164618.1

AF164617.1

AH008411.1

AF164621.1

AF164620.1

AH008412.1

AF164616.1

AF164619.1

AH008413.1

AF081546.1

AF081547.1

AF081548.1

AF081549.1

AF081550.1

AF081551.1

AF133658.1

AF134163.1

AF134164.1

AF205860.1

NM_014383.1

AF156961.1

AF156962.1

AF156963.1

AF216972.1

AF074086.2

AF264058.1

AB047241.1

AB047242.1

AJ297040.1

AF261945.1

AF290421.1

AF290422.1

AF290423.1

AB043907.1

AB043908.1

AB043909.1

AB043910.1

AB043911.1

AB043912.1

AB043913.1

AB043914.1

AB043915.1

AB043916.1

AB043917.1

AB043918.1

AB043919.1

AB043920.1

AB043921.1

AB043922.1

AB043923.1

AB043924.1

AB043925.1

AB043926.1

AB043927.1

AB043928.1

AB043929.1

AB043930.1

AB043931.1

AB043932.1

AB043933.1

AB043934.1

AB043935.1

AB043936.1

AB043937.1

AB043938.1

AB043939.1

AB043940.1

AB043941.1

AB043942.1

AF315797.1

AF315798.1

AF315799.1

AF315800.1

AF315801.1

AF298587.1

AF298588.1

AF239665.1

AX092223.1

AX092224.1

AX092225.1

AX092226.1

AX092227.1

AX092228.1

AX092229.1

AX092230.1

AX092231.1

AX092232.1

AX092233.1

AX092234.1

AX092235.1

AX092236.1

AX092237.1

AX092238.1

AX092239.1

AX092240.1

AX092241.1

AX092242.1

AX092243.1

AX092244.1

AX092245.1

AX092246.1

AX092247.1

AX092248.1

AX092249.1

AB050996.1

AB050997.1

AB050998.1

AB050999.1

AB051000.1

AB051001.1

AB051002.1

AB051003.1

AB051004.1

AB051005.1

AB051006.1

AB051007.1

AB051008.1

AB051009.1

AB051010.1

AB051564.1

AB051565.1

AB051566.1

AB051567.1

AB051568.1

AB051569.1

AB051570.1

AB051571.1

NM_032498.1

AF370125.1

AB052567.1

AB052568.1

AB052569.1

AB052570.1

AB052571.1

AB052572.1

AB052573.1

AB052574.1

AB052575.1

AB052576.1

AB054085.1

AB054089.1

AB054086.1

AB054087.1

AB054088.1

AF394944.1

AB055412.1

AB055413.1

AB055414.1

AB055415.1

AB055416.1

AB055868.1

AB055869.1

AB055870.1

AB055871.1

AB055872.1

AB055873.1

AB055874.1

AB055875.1

AB055876.1

AB055877.1

AB055878.1

AB055879.1

AY050297.1

AY050298.1

AB060002.1

AB060003.1

AB060004.1

AB060005.1

AB060006.1

AB060007.1

AB060008.1

AB060009.1

AB060010.1

AB060011.1

AB060012.1

AB060013.1

AB060014.1

AB060015.1

AB060016.1

AB060017.1

AB060018.1

AB060019.1

AB060020.1

AB060021.1

AB060022.1

AB060023.1

AB060024.1

AB060025.1

AB060026.1

AB060027.1

AB060028.1

AB060029.1

AB060030.1

AB060031.1

AB060032.1

AB060033.1

AB060034.1

AB060035.1

AB060036.1

AB060037.1

AB060038.1

AB060039.1

AB060040.1

AB060041.1

AB060042.1

AB060043.1

AB060044.1

AB060045.1

AB060046.1

AB060047.1

AB060048.1

AB060049.1

AB060050.1

AB062072.1

AB062073.1

AB062074.1

AB062075.1

AB062076.1

AB062077.1

AB062078.1

AB062274.1

AB063618.1

AB063619.1

AB063620.1

AB063621.1

AK074464.1

AJ431196.1

AJ431197.1

NG_001298.1

AY098483.1

AY098482.1

AF480924.1

AF531174.1

AY070774.1

AY070775.1

AY186778.1

AY208132.1

AY208133.1

AY208134.1

AY208135.1

AY208136.1

BL000002.1

AF499232.1

AB096021.1

AB096022.1

AB096023.1

AB096024.1

AB096025.1

AB096026.1

AB096027.1

AB096028.1

AB096029.1

AB096030.1

AB096031.1

AB096032.1

AB096033.1

AB096034.1

AB096035.1

AB096036.1

AB096037.1

AB096038.1

AY224378.1

AB100173.1

AB100174.1

AB100175.1

AB100176.1

AB100177.1

AB100178.1

AB100179.1

AB100180.1

AB100181.1

AB100182.1

AB100183.1

AB100184.1

AB100185.1

AB100186.1

AB100187.1

AB100188.1

AB100189.1

AB100190.1

AB100191.1

AB100192.1

AB100193.1

AB100194.1

AB100195.1

AB100196.1

AB100197.1

AB100198.1

AB100199.1

AB100200.1

AB100201.1

AB100202.1

AB100203.1

AB100204.1

AB100205.1

AB100206.1

AB100207.1

AB100208.1

AB100209.1

AB100210.1

AB100211.1

AB100212.1

AB100213.1

AB100214.1

AB100215.1

AB100216.1

AB100217.1

AB100218.1

AB100219.1

AB100220.1

AB100221.1

AB100222.1

AB100223.1

AB100224.1

AB100225.1

AB100226.1

AB100227.1

AB100228.1

AB100229.1

AB100230.1

AB100231.1

AB100232.1

AB100233.1

AB100234.1

AB100235.1

AB100236.1

AB100237.1

AB100238.1

AB100239.1

AB100240.1

AB100241.1

AB100242.1

BD223751.1

BD223752.1

BD223753.1

BD223754.1

BD223755.1

BD223756.1

BD223757.1

AK123566.1

AK124077.1

AK124340.1

AY371029.1

AY371030.1

AY371031.1

AY371032.1

AY371033.1

AY371034.1

AY371035.1

AY371036.1

AY371037.1

AY371038.1

AY371039.1

AY371040.1

AY371041.1

AY371042.1

AY371043.1

AY371044.1

AY371045.1

AY371046.1

AY395517.1

AY395518.1

AY395519.1

AY395520.1

AY395521.1

AY395522.1

AY395523.1

AY395524.1

AY395525.1

AY395526.1

AY101582.1

AY101583.1

AY101584.1

AY101585.1

L36092.2

AB128832.1

NR_001591.1

AB114916.1

AB114917.1

AB114918.1

AB114919.1

AB114920.1

AB114921.1

AB114922.1

AB114923.1

AB114924.1

AB114925.1

AB114926.1

AB114927.1

AB114928.1

AB114929.1

AB114930.1

AB114931.1

AB114932.1

AB114933.1

AB114934.1

AB114935.1

AB114936.1

AB114937.1

AB114938.1

AB114939.1

AB114940.1

AB114941.1

AB114942.1

AB114943.1

AB114944.1

AB114945.1

AB114946.1

AB114947.1

AB114948.1

AB114949.1

AB114950.1

AB114951.1

AB114952.1

AB114953.1

AB114954.1

AB114955.1

AB114956.1

AB114957.1

AB114958.1

AB114959.1

AB114960.1

AB114961.1

AB114962.1

AB114963.1

AB114964.1

AB114965.1

AB114966.1

AB114967.1

AB114968.1

AB114969.1

AB114970.1

AB114971.1

AB114972.1

AB114973.1

AB114974.1

AB114975.1

AB114976.1

AB114977.1

AB114978.1

AB114979.1

AB114980.1

AB114981.1

AB114982.1

AB114983.1

AB114984.1

AB114985.1

AB114986.1

AB114987.1

AB114988.1

AB114989.1

AB114990.1

AB114991.1

AB114992.1

AB114993.1

AB114994.1

AB114995.1

AB114996.1

AB114997.1

AB114998.1

AB114999.1

AB115000.1

AB115001.1

AB115002.1

AB115003.1

AB115004.1

AB115005.1

AB115006.1

AB115007.1

AB115008.1

AB115009.1

AB115010.1

AB115094.1

AB115095.1

AB115096.1

AB115097.1

AB115098.1

AB115099.1

AB115100.1

AB115101.1

AB115102.1

AB115103.1

AB115104.1

AB115105.1

AB115106.1

AB115107.1

AB115108.1

AB115109.1

AB115110.1

AB115111.1

AB115112.1

AB115113.1

AB115114.1

AB115115.1

AB115116.1

AB115117.1

AB115118.1

AB115119.1

AB115120.1

AB115121.1

AB115122.1

AB115123.1

AB115124.1

AB115125.1

AB115126.1

AB115127.1

AB115128.1

AB115129.1

AB115130.1

AB115131.1

AB115132.1

AB115133.1

AB115134.1

AB115135.1

AB115136.1

AB115137.1

AB115138.1

AB115139.1

AB115140.1

AB115141.1

AB115142.1

AB115143.1

AB115144.1

AB115145.1

AB115146.1

AB115147.1

AB115148.1

AB115149.1

AB115150.1

AB115151.1

AB115152.1

AB115153.1

AB115154.1

AB115155.1

AB115156.1

AB115157.1

AB115158.1

AB115159.1

AB115160.1

AB115161.1

AB115162.1

AB115163.1

AB115164.1

AB115165.1

AB115166.1

AB115167.1

AB115168.1

AB115169.1

AB115170.1

AB115171.1

AB115172.1

AB115173.1

AB115174.1

AB115175.1

AB115176.1

AB115177.1

AB115178.1

AB115186.1

AB115187.1

AB115188.1

AB115189.1

AB115190.1

AB115191.1

AB115192.1

AB115193.1

AB115194.1

AB115195.1

AB115196.1

AB115197.1

AB115198.1

AB115199.1

AB115200.1

AB115201.1

AB115202.1

AB115203.1

AB115204.1

AB115205.1

AB115206.1

AB115207.1

AB115208.1

AB115209.1

AB115210.1

AB115211.1

AB115212.1

AB115213.1

AB115214.1

AB115215.1

AB115216.1

AB115217.1

AB115218.1

AB115219.1

AB115220.1

AB115221.1

AB115222.1

AB115223.1

AB115224.1

AB115225.1

AB115226.1

AB115227.1

AB115228.1

AB115229.1

AB115230.1

AB115231.1

AB115232.1

AB115233.1

AB115234.1

AB115235.1

AB115236.1

AB115237.1

AB115238.1

AB115247.1

AB115248.1

AB115249.1

AB115250.1

AB115251.1

AB115252.1

AB115253.1

AB115254.1

AB115255.1

AB115256.1

AB115257.1

AB115258.1

AB115259.1

AB115260.1

AB115261.1

AB115262.1

AB115263.1

AB115264.1

AB115265.1

AB115266.1

AB115267.1

AB115268.1

AB115269.1

AB115270.1

AB115271.1

AB115272.1

AB115273.1

AB115274.1

AB115275.1

AB115276.1

AB115277.1

AB115278.1

AB115279.1

AB115280.1

AB115281.1

AB115282.1

AB115283.1

AB115284.1

AB115285.1

AB115286.1

AB115287.1

AB115288.1

AB115289.1

AB115290.1

AB115291.1

AB115292.1

AB115293.1

AB115294.1

AB115295.1

AB115296.1

AB115297.1

AB115298.1

AB115299.1

AB115300.1

AB115301.1

AB115302.1

AB115303.1

AB115304.1

AB115305.1

AB115306.1

AY541703.1

AY541704.1

AB120770.1

AB120771.1

AB120772.1

AB120773.1

AB120774.1

AB120775.1

AB120776.1

AB120777.1

AB120778.1

AB120779.1

AB120780.1

AB120781.1

AB120890.1

AB120891.1

AB120892.1

AB120893.1

AB120894.1

AB120895.1

AB120896.1

AB120897.1

AB120898.1

AB120899.1

AB120900.1

AB120901.1

AB120902.1

AB120903.1

AB120904.1

AB120905.1

AB120906.1

AB120907.1

AB120908.1

AB120909.1

AB120910.1

AB120911.1

AB120912.1

AB120913.1

AB120914.1

AB120915.1

AB120916.1

AB120917.1

AB120918.1

AB120919.1

AB120920.1

AB120921.1

AB120922.1

AB120923.1

AB120924.1

AB120925.1

AB120926.1

AB120927.1

AB120928.1

AB120929.1

AB120930.1

AB120931.1

AB120932.1

AB120933.1

AB120934.1

AB120935.1

AB120936.1

AB120937.1

NM_005867.2

NM_014590.3

BL000001.2

AB167270.1

AB167271.1

AB167272.1

AB167273.1

AB167274.1

AB167275.1

AB167276.1

AB167277.1

AB167278.1

AB167279.1

AB167280.1

AB167281.1

AB167282.1

AB167283.1

AB167284.1

AB167285.1

AB167286.1

AB167287.1

AB167288.1

AB167289.1

AB167290.1

AB167291.1

AB167292.1

AB167293.1

AB167294.1

AB167295.1

AB167296.1

AB167297.1

AB167298.1

AB167299.1

AB167300.1

AB167301.1

AB167302.1

AB167303.1

AB167304.1

AB167305.1

AB167306.1

AB167307.1

AB167308.1

AB167309.1

AB167310.1

AB167311.1

AB167312.1

AB167313.1

AB167314.1

AB167315.1

AB167316.1

AB167317.1

AB167318.1

AB167319.1

AB167320.1

AB167321.1

AB167322.1

AB167323.1

AB167324.1

AB167325.1

AB167326.1

AB167327.1

AB167328.1

AB167329.1

AB167330.1

AB167331.1

AB167332.1

AB167333.1

AB167334.1

AB167335.1

AB167336.1

AB167337.1

AB167338.1

AB167339.1

AB167340.1

AB167341.1

AB167342.1

AB167343.1

AB167344.1

AB167345.1

AB167346.1

AB167347.1

AB167348.1

CQ890993.1

AY920278.1

AY920279.1

AY920280.1

AY920281.1

DQ058013.1

DQ058014.1

DQ058015.1

DQ058016.1

CH471135.1

DQ112093.1

DQ112094.1

DQ112095.1

DQ112096.1

DQ112097.1

DQ112098.1

DQ112099.1

DQ112100.1

DQ112101.1

DQ112102.1

DQ112103.1

DQ112104.1

DQ112105.1

DQ112106.1

DQ112107.1

DQ112108.1

DQ112109.1

DQ112110.1

DQ112111.1

DQ112113.1

DQ112114.1

DQ112115.1

DQ112116.1

DQ112117.1

DQ112118.1

DQ112119.1

DQ112120.1

DQ112121.1

DQ112122.1

DQ112123.1

DQ112124.1

DQ112127.1

DQ112131.1

DQ112132.1

DQ112134.1

DQ112139.1

DQ112140.1

DQ112143.1

DQ112146.1

DQ112147.1

DQ112148.1

DQ112151.1

DQ112152.1

CM000270.1

DQ179113.1

DQ247958.1

NM_004602.2

NM_017452.2

NM_017453.2

NM_017454.2

NM_001037328.1

BD297017.1

BD297018.1

BD297019.1

BD297020.1

BD297021.1

BD297022.1

BD297023.1

BD297024.1

BD297025.1

BD297026.1

BD297027.1

BD297028.1

BD297029.1

BD297030.1

BD297031.1

BD297032.1

BD297033.1

BD297034.1

BD297035.1

BD297036.1

BD297037.1

BD297038.1

BD297039.1

BD297040.1

BD297041.1

BD297042.1

BD297043.1

AB239999.1

AB240000.1

AB240001.1

AB240002.1

AB240003.1

AB240004.1

AB240005.1

AB240006.1

AB240007.1

AB240008.1

AB240009.1

AB240010.1

AB240011.1

AB240012.1

AB240013.1

AB240014.1

AB240015.1

AB240016.1

AB240017.1

AB240018.1

AB240019.1

AB240020.1

AB240021.1

AB240022.1

AB240023.1

AB240024.1

AB240025.1

AB240026.1

AB240027.1

AB240028.1

AB240029.1

AB240030.1

AB240031.1

AB240032.1

AB240033.1

AB240034.1

AB240035.1

AB240036.1

AB240037.1

AB240038.1

AB240039.1

AB240040.1

AB240041.1

AB240042.1

AB240043.1

AB240044.1

AB240045.1

AB259217.1

AB259218.1

AB259219.1

AB259220.1

AB259221.1

AB259222.1

AB259223.1

AB259224.1

AB259225.1

AB259226.1

AB259227.1

AB259228.1

AB259229.1

AB259230.1

AB259231.1

AB259232.1

AB259233.1

AB259234.1

AB259235.1

AB259236.1

AB259237.1

AB259238.1

AB259239.1

AB259240.1

AB259241.1

AB259242.1

AB259243.1

AB259244.1

AB259245.1

AB259246.1

AB259247.1

AB259248.1

AB259249.1

AB259250.1

AB259251.1

AB259252.1

AB259253.1

AB259254.1

AB259255.1

AB259256.1

AB259257.1

AB259258.1

AB259259.1

AB259260.1

AB259261.1

AB259262.1

AB259263.1

AB259264.1

AB259265.1

AB259266.1

AB259267.1

AB259268.1

AB259269.1

AB259270.1

AB259271.1

AB259272.1

AB259273.1

AB259274.1

AB259275.1

AB259276.1

AB259277.1

AB259278.1

AB259279.1

AB259280.1

AB259281.1

AB259282.1

AB259283.1

AB259284.1

AB259285.1

AB259286.1

EF194101.1

EF535612.1

EF535613.1

NG_003135.2

NM_001104577.1

NM_017986.3

NW_001838913.1

NW_001838973.1

NW_001839034.1

NW_001839072.1

NW_001839223.1

NW_001838341.1

AC_000133.1

AC_000148.1

AC_000151.1

AC_000136.1

AC_000138.1

AC_000139.1

AC_000141.1

NW_001838553.2

NW_001839064.2

NW_001838498.2

EU195218.1

EU195219.1

EU195220.1

EU195221.1

AB266802.1

NM_001470.2

NM_021903.2

NM_021904.2

NM_032622.2

EU669866.1

EU791617.1

AK303822.1

NM_001130925.1

AB443932.1

AB443933.1

AB443934.1

AB443935.1

AB443936.1

AB443937.1

EU853142.1

EU853143.1

NM_016255.2

NG_009310.1

NG_009311.1

NM_001080399.2

NM_001145095.1

NM_001145805.1

DM171307.1

DM171308.1

DM171309.1

DM171310.1

DM171311.1

DM171312.1

DM171313.1

DM171314.1

DM171315.1

DM171316.1

DM171317.1

DM171318.1

DM171319.1

DM171320.1

DM171321.1

DM171322.1

DM171323.1

DM171324.1

DM171325.1

DM171326.1

DM171327.1

DM171328.1

DM171329.1

DM171330.1

DM171331.1

DM171332.1

DM171333.1

NM_207582.2

NR_030368.1

NR_030386.1

NM_153189.2

NM_003117.4

NM_001174044.1

NM_001174045.1

NM_001174046.1

FN689795.1

HC480402.1

HC480403.1

HC480404.1

NM_001007253.3

NM_152473.2

NM_001191055.1

NM_002249.5

NM_170782.2

NM_001204087.1

NG_001299.2

JF262036.1

JF262037.1

NG_028722.1

FR714893.1

FR714894.1

FR714895.1

FR714896.1

FR714897.1

FR714898.1

JF960161.1

JF960162.1

JF960163.1

JF960164.1

NM_024534.5

NM_001242690.1

NR_040023.1

AH004271.2

JN675098.1

JN675099.1

JN675100.1

JN675101.1

JN675102.1

JN675103.1

JN675104.1

JN675105.1

JN675106.1

JN675107.1

JN675108.1

JN675109.1

JN675110.1

JN675111.1

JN675112.1

JN675113.1

JN675114.1

JN675115.1

JN675116.1

JN675117.1

JN675118.1

JN675119.1

JN675120.1

JN675121.1

JN675122.1

JN675123.1

JN675124.1

JN675125.1

JN675126.1

JN675127.1

JN675128.1

JN675129.1

JN675130.1

JN675131.1

JN675132.1

JN675133.1

JN675134.1

JN675135.1

JN675136.1

JN675137.1

JN675138.1

JN675139.1

JN675140.1

JN675141.1

JN675142.1

JN675143.1

JN675144.1

JN675145.1

JN675146.1

JN675147.1

JN675148.1

JN675149.1

JN675150.1

JN675151.1

JN675152.1

JN675153.1

JN675154.1

JN675155.1

JN675156.1

JN675157.1

JN675158.1

JN675159.1

JN675160.1

JN675161.1

JN675162.1

JN675163.1

JN675164.1

JN675165.1

JN675166.1

JN675167.1

JN675168.1

JN675169.1

JN675170.1

JN675171.1

JN675172.1

JN675173.1

JN675174.1

JN675175.1

JN675176.1

JN675177.1

JN675178.1

JN675179.1

JN675180.1

JN675181.1

JN675182.1

JN675183.1

JN675184.1

JN675185.1

JN675186.1

JN675187.1

JA662133.1

NM_024531.4

NM_001253815.1

NM_001253816.1

NR_045600.1

NM_001256030.1

NM_001778.3

NM_001126328.2

NG_032704.1

NG_032725.1

AB728590.1

AB728591.1

KC010496.1

KC010508.1

NC_018930.2

NC_018927.2

NC_018920.2

NC_018918.2

NC_018917.2

NC_018915.2

NC_018912.2

NT_011109.17

NT_010393.17

NT_008470.20

NT_007933.16

NT_007592.16

NT_022853.16

NT_167186.2

NC_000019.10

NC_000016.10

NC_000009.12

NC_000007.14

NC_000006.12

NC_000004.12

NC_000001.11

AJ233665.1

AJ233666.1

AJ233667.1

AJ233668.1

AJ233669.1

XM_002922068.1

X51929.1

AF164916.1

AF164917.1

AF164919.1

AF164920.1

M21156.1

M21157.1

M21158.1

AJ233664.1

AF164912.1

AF164913.1

AF164921.1

AF164922.1

AF164923.1

EU153401.1

AB674439.1

AB674440.1

AB674441.1

AB674442.1

AB674443.1

AB674444.1

AB674445.1

AB674446.1

AB674447.1

AB674448.1

AB674449.1

AB674450.1

AB674451.1

AB674452.1

AB674453.1

AB674572.1

AB674573.1

AB674574.1

AB674575.1

AB674576.1

AB674577.1

NW_004064727.1

NC_018727.1

AB679509.1

JX406428.1

JX406429.1

GQ222416.1

AF312188.1

AF312189.1

AF312190.1

AF312191.1

AF312192.1

AF312193.1

AF312194.1

AF312195.1

AF312196.1

AF312197.1

AY394590.1

AY394591.1

AY394592.1

AY394593.1

AY394594.1

AY394595.1

AY394596.1

AY394597.1

AY394598.1

AY394599.1

AF317797.1

AF317798.1

AF312128.1

AF312129.1

AF312130.1

AF312131.1

AF312132.1

AF312133.1

AF312134.1

AF312135.1

AF312136.1

AF312137.1

AF312138.1

AF312139.1

AF312140.1

AF312141.1

AF312142.1

AF312143.1

AF312144.1

AF312145.1

AF312146.1

AF312147.1

AF312148.1

AF312149.1

AF312150.1

AF312151.1

AF312152.1

AF312153.1

AF312154.1

AF312155.1

AF312156.1

AF312157.1

AY394571.1

AY394572.1

AY394573.1

AY394574.1

AY394575.1

AY394576.1

AY394577.1

AY394578.1

AY394579.1

AF317799.1

AF317800.1

AF312158.1

AF312159.1

AF312160.1

AF312161.1

AF312162.1

AF312163.1

AF312164.1

AF312165.1

AF312166.1

AF312167.1

AF312168.1

AF312169.1

AF312170.1

AF312171.1

AF312172.1

AF312173.1

AF312174.1

AF312175.1

AF312176.1

AF312177.1

AF312178.1

AF312179.1

AF312180.1

AF312181.1

AF312182.1

AF312183.1

AF312184.1

AF312185.1

AF312186.1

AF312187.1

AY394580.1

AY394581.1

AY394582.1

AY394583.1

AY394584.1

AY394585.1

AY394586.1

AY394587.1

AY394588.1

AY394589.1

AJ233650.1

AJ233651.1

AJ233652.1

AJ233653.1

AJ233654.1

AJ233655.1

AJ233656.1

AF312198.1

AF312199.1

AF312200.1

AF312201.1

AF312202.1

AF312203.1

AF312204.1

AF312205.1

AF312206.1

AF312207.1

AY394600.1

AY394601.1

AY394602.1

AY394603.1

AY394604.1

AY394605.1

AY394606.1

AY394607.1

AY394608.1

AY394609.1

U77599.1

AF021931.1

AF021932.1

AF021933.1

AF021934.1

AF038599.1

AF038600.1

AF038601.1

AF296168.1

AF277320.1

AF277321.1

AF277322.1

AF274705.1

AF274706.1

AF274707.1

AF274708.1

AF274709.1

AF274710.1

AF274711.1

AF274712.1

AF274713.1

AY056024.1

AY056025.1

AY056026.1

AY056027.1

AY056028.1

AF426916.1

AF426917.1

AF426918.1

AF426919.1

AF426920.1

AF426921.1

AF426922.1

AF426923.1

AF426924.1

AF426925.1

AF426926.1

AF426927.1

AF426928.1

AF426929.1

AF426930.1

AF426931.1

AF426932.1

AF426933.1

AF426934.1

AF426935.1

AF426936.1

AF426937.1

AF426938.1

AF426939.1

AF426940.1

AF426941.1

AF426942.1

AF426943.1

AF426944.1

AF426945.1

AF426946.1

AF511088.1

AF511089.1

AF511090.1

AF511091.1

AF511092.1

AF511093.1

AF511094.1

AF511095.1

AF511096.1

AF511097.1

AF511098.1

AF511099.1

AF511100.1

AF511101.1

AF511102.1

AF511103.1

AF511104.1

AF511105.1

AF511106.1

AF511107.1

AF511108.1

AF511109.1

AF511110.1

AF511111.1

AF511112.1

AF511113.1

AF511114.1

AF511115.1

AJ303092.1

AH012445.1

AY160111.1

AY160112.1

AH012446.1

AY160113.1

AY160114.1

AY134475.1

AY288779.1

AY312517.1

AY312518.1

AY312519.1

AY312520.1

AY312521.1

AY312522.1

AY312523.1

AY312524.1

AY312525.1

AY312526.1

AY312527.1

AY312528.1

AY312529.1

AY312530.1

AY312531.1

AY312532.1

AY312533.1

AY312534.1

AY312535.1

AY312536.1

AY312537.1

AY312538.1

AY312539.1

AY312540.1

AY312541.1

AY312542.1

AY312543.1

AY312544.1

AY312545.1

AY312546.1

AY312547.1

AY312548.1

AY312549.1

AY312550.1

AY312551.1

AY312552.1

AY312553.1

AY312554.1

AY312555.1

AY312556.1

AY312557.1

AY312558.1

AY312559.1

AY312560.1

AY312561.1

AY312562.1

AY312563.1

AY312564.1

AY312565.1

AY312566.1

AY312567.1

AY394852.1

AJ304824.2

NM_001004033.1

DQ011693.1

DQ011694.1

DQ011695.1

DQ011696.1

DQ011697.1

DQ011698.1

DQ011699.1

DQ011700.1

DQ011701.1

DQ011702.1

DQ011703.1

DQ011704.1

DQ011705.1

DQ011706.1

DQ011707.1

DQ011708.1

DQ011709.1

DQ011710.1

DQ011711.1

DQ011712.1

DQ011713.1

DQ011714.1

DQ011715.1

DQ011716.1

DQ011717.1

DQ011718.1

DQ011719.1

DQ011720.1

DQ011721.1

DQ011722.1

DQ011723.1

DQ011724.1

DQ011725.1

DQ011726.1

DQ011727.1

DQ011728.1

DQ011729.1

DQ011730.1

DQ011731.1

DQ011732.1

DQ011733.1

DQ011734.1

DQ011735.1

DQ011736.1

DQ011737.1

DQ011738.1

DQ011739.1

DQ011740.1

DQ011741.1

DQ011742.1

DQ011743.1

DQ011744.1

DQ011745.1

DQ011746.1

DQ011747.1

DQ011748.1

DQ011749.1

DQ011750.1

DQ011751.1

DQ011752.1

DQ011753.1

DQ011754.1

DQ011755.1

DQ011756.1

DQ011757.1

DQ011758.1

DQ011759.1

DQ011760.1

DQ011761.1

DQ011762.1

DQ011763.1

DQ011764.1

DQ011765.1

DQ011766.1

DQ011767.1

DQ011768.1

DQ011769.1

DQ011770.1

DQ011771.1

DQ011772.1

DQ011773.1

DQ011774.1

DQ011775.1

DQ011776.1

DQ011777.1

DQ011778.1

DQ011779.1

DQ011780.1

DQ011781.1

DQ011782.1

DQ011783.1

DQ011784.1

DQ011785.1

DQ011786.1

DQ011787.1

DQ011788.1

DQ011789.1

DQ011790.1

DQ011791.1

DQ011792.1

DQ011793.1

DQ011794.1

DQ011795.1

DQ011796.1

DQ011797.1

DQ011798.1

DQ011799.1

DQ011800.1

DQ011801.1

DQ011802.1

DQ011803.1

DQ011804.1

DQ011805.1

DQ011806.1

DQ011807.1

DQ011808.1

DQ011809.1

DQ011810.1

DQ011811.1

DQ011812.1

DQ011813.1

DQ011814.1

DQ011815.1

DQ011816.1

DQ011817.1

DQ011818.1

DQ011819.1

DQ011820.1

DQ011821.1

DQ011822.1

DQ011823.1

DQ011824.1

DQ011825.1

DQ011826.1

DQ011827.1

DQ011828.1

DQ011829.1

DQ011830.1

DQ011831.1

DQ011832.1

DQ011833.1

DQ011834.1

DQ084470.1

DQ084471.1

DQ084472.1

DQ084473.1

DQ084474.1

DQ084475.1

DQ084476.1

DQ084477.1

DQ084478.1

DQ084479.1

DD161074.1

DD161075.1

DD161076.1

DQ650703.1

DQ650704.1

DQ835391.1

DQ835392.1

DQ835393.1

AM229311.1

AM229313.1

DQ996273.1

EF185173.1

EF185174.1

EF185202.1

NM_001097446.1

EU789636.1

GQ906168.1

GQ906169.1

GQ906201.1

GQ906202.1

GQ906229.1

GQ906230.1

GQ906231.1

GQ906232.1

GQ906269.1

GQ906270.1

GQ906271.1

GQ906283.1

GQ906284.1

GQ906285.1

GQ906286.1

GQ906317.1

GQ906318.1

GQ906319.1

GQ906320.1

EU871586.1

HM347451.1

AB535570.1

AB535571.1

AB535572.1

AB535573.1

AB535574.1

AB535575.1

AB535576.1

AB535577.1

AB535578.1

AB535579.1

AB535580.1

AB535581.1

AB535582.1

AB535583.1

AB535584.1

AB535585.1

AB535586.1

AB535587.1

AB535588.1

AB535589.1

AB535590.1

AB535591.1

AB535592.1

AB535593.1

AB535594.1

AB535595.1

AB535596.1

AB535597.1

AB535598.1

HQ456148.1

HQ456149.1

HQ456150.1

HQ456151.1

HQ456182.1

HQ456183.1

HQ456184.1

HQ456185.1

HQ456186.1

FJ716801.2

FJ716802.2

HQ536005.1

HQ536006.1

HQ536007.1

HQ536008.1

HQ536009.1

HQ536010.1

HQ536011.1

HQ536012.1

HQ536013.1

HQ536014.1

HQ536015.1

HQ536016.1

NW_003611203.1

NC_010451.3

AB645931.1

AB645932.1

AB645933.1

AB645934.1

AB645935.1

AB645936.1

AB645937.1

AB645938.1

AB645939.1

AB645940.1

AB645941.1

AB645942.1

AB645943.1

AB645944.1

AB645945.1

AB645946.1

AB645947.1

AB645948.1

AB645949.1

AB645950.1

AB645951.1

AB645952.1

AB645953.1

AB645954.1

AB645955.1

AM229312.2

KC116218.1

KC116219.1

KC116220.1

KC116221.1

U77600.1

AJ233661.1

AF147808.1

BD375893.1

BD375894.1

BD375895.1

BD375896.1

BD375897.1

BD375898.1

BD375899.1

BD375900.1

BD375901.1

BD375902.1

BD375903.1

BD375904.1

BD375905.1

BD375906.1

BD375907.1

BD375908.1

JN592050.1

AB749813.1

AJ233662.1

AJ233663.1

EF030818.1

AB510398.1

AB587259.1

AB587260.1

AB753777.1

AB749816.1

AB749817.1

AB749818.1

AB751366.1

AY193894.1

AY193895.1

AY193896.1

AY193897.1

AY193898.1

AY193899.1

AY193900.1

AY193901.1

AY193902.1

AY193903.1

AY193904.1

AY193905.1

AY193906.1

AY193907.1

AY193908.1

AY193909.1

AY266331.1

AY266332.1

AJ233625.1

AJ233626.1

AJ233627.1

AF480925.1

AF480926.1

AF480927.1

AF480928.1

AF480929.1

AF515800.1

NW_003161034.1

NW_003160827.1

NW_003160146.1

NW_003159943.1

NW_003159517.1

NW_003159405.1

NW_003159396.1

NW_003159395.1

NW_003159376.1

NW_003159331.1

NW_003159327.1

NW_003159326.1

NW_003159324.1

NW_003159302.1

NW_003159300.1

NW_003159299.1

NW_003159292.1

NW_003159282.1

NW_003159276.1

NW_003159230.1

NW_003159229.1

NC_013690.1

NC_013685.1

NC_013684.1

NC_013682.1

NC_013680.1

NC_013679.1

NC_013669.1

X52271.1

NW_003616633.1

NW_003616169.1

NW_003615557.1

NW_003613944.1

NW_003613588.1

DQ139733.1

DQ139734.1

DQ139735.1

DQ139736.1

DQ139737.1

DQ139738.1

DQ139739.1

DQ139740.1

DQ139741.1

DQ139742.1

DQ139743.1

DQ139744.1

DQ139745.1

DQ139746.1

DQ139748.1

DQ139749.1

DQ139750.1

DQ139751.1

DQ139752.1

DQ139753.1

DQ139754.1

DQ139755.1

DQ139756.1

M63645.1

M63646.1

M63647.1

M63643.1

M63644.1

X52622.1

X16669.1

X16670.1

X16671.1

X16672.1

X00433.1

M15116.1

M15117.1

AH001845.1

M10062.1

K03230.1

M11301.1

M12315.1

M11051.1

M11052.1

AH001894.1

M33884.1

M12313.1

M12312.1

K02410.1

K02411.1

K02412.1

K02413.1

K02414.1

M15369.1

K00013.1

K00014.1

AH001896.1

M12314.1

M10108.1

M10109.1

M26005.1

M26006.1

K02892.1

M12785.1

M12786.1

AH002036.1

M29071.1

M29070.1

M27506.1

M27505.1

AH002045.1

M13187.1

M13188.1

X75261.1

U06639.1

X72930.1

L37058.1

L37057.1

U63133.1

Y12713.1

AC004093.1

AC004155.1

AJ233590.1

AJ233591.1

AJ233592.1

AJ233593.1

AJ233594.1

AJ230107.1

AJ230108.1

AJ230109.1

AJ230110.1

AJ230111.1

AJ230112.1

AJ230113.1

AJ230114.1

AJ230115.1

AJ230116.1

AJ230117.1

AJ230118.1

AJ230119.1

AJ230120.1

AJ230121.1

AJ230122.1

AJ230123.1

AJ230124.1

AF093696.1

AF093697.1

AF093698.1

AF093699.1

AF093700.1

AF093701.1

AF246632.1

AF246633.1

AF246129.1

AF246130.1

AK014027.1

AF295923.1

AF289866.1

AF329434.1

AY061809.1

AY061810.1

AY061811.1

AY608536.1

AY608537.1

AY608538.1

AY608539.1

AY608540.1

AY608541.1

AY608542.1

AY608543.1

AY608544.1

AY608545.1

AY608546.1

AY608547.1

AY608548.1

AY608549.1

AY608550.1

AY608551.1

AY608552.1

AY608553.1

AY608554.1

AY608555.1

AY608556.1

AY608557.1

AY608558.1

AY608559.1

AY608560.1

AY608561.1

AY608562.1

AY608563.1

AY608564.1

AY608565.1

AY608566.1

AY608567.1

AY608568.1

AY608569.1

AY608570.1

AY608571.1

AY608572.1

AY608573.1

AY608574.1

AY608575.1

AY920282.1

AY920283.1

AY934758.1

DQ157737.1

DQ157738.1

DQ157739.1

DQ157740.1

DQ366147.1

DQ366148.1

NM_007910.2

NM_009201.2

AB060642.1

AB060643.1

NM_019743.3

NM_007777.3

NM_026385.4

NM_012031.3

EU118153.1

EU118154.1

NM_017391.3

EU024536.1

NM_033149.3

NM_001122993.1

EU636232.1

FJ654066.1

FJ654067.1

FJ654068.1

FJ654069.1

FJ654070.1

FJ654071.1

FJ654072.1

FJ654073.1

FJ654074.1

FJ654075.1

FJ654076.1

FJ654077.1

FJ654078.1

FJ654079.1

FJ654080.1

FJ654081.1

FJ654082.1

FJ654083.1

FJ654084.1

FJ654085.1

FJ654086.1

FJ654087.1

FJ654088.1

FJ654089.1

FJ654090.1

FJ654091.1

FJ654092.1

FJ654093.1

FJ654094.1

FJ654095.1

FJ654096.1

FJ654097.1

FJ654098.1

FJ654099.1

FJ654100.1

FJ654101.1

FJ654102.1

FJ654103.1

FJ654104.1

FJ654105.1

FJ654106.1

FJ654107.1

FJ654108.1

FJ654109.1

FJ654110.1

FJ654111.1

FJ654112.1

FJ654113.1

FJ654114.1

FJ654115.1

FJ654116.1

FJ654117.1

FJ654118.1

FJ654119.1

FJ654120.1

FJ654121.1

FJ654122.1

FJ654123.1

FJ654124.1

FJ654125.1

FJ654126.1

FJ654127.1

FJ654128.1

FJ654129.1

FJ654130.1

FJ654131.1

FJ654132.1

FJ654133.1

FJ654134.1

FJ654135.1

NM_029662.2

NM_018861.3

NM_173420.3

HE599401.1

AY999005.1

M23459.1

AJ233670.1

AJ233671.1

X16839.1

U60098.1

D90005.1

AY212271.2

AY769433.1

NM_001024239.1

NW_001084749.1

NW_001084766.1

NW_001084813.1

NW_001084835.1

NW_001084845.1

NW_001084854.1

NW_001084856.1

NW_001084859.1

NW_001084659.1

NW_001084670.1

NW_001084679.1

NW_001084681.1

NW_001084686.1

NW_001084709.1

NW_001084715.1

NW_001084737.1

NW_001091918.1

NW_001091920.1

NW_001091925.1

NW_001084892.1

NW_001084906.1

NW_001084921.1

NW_001085134.1

AC_000069.1

AC_000079.1

AC_000080.1

AC_000081.1

AC_000083.1

AC_000084.1

AC_000086.1

AC_000071.1

AC_000073.1

AC_000074.1

AC_000075.1

AC_000089.1

EF532341.1

NC_005120.4

NC_005116.4

NC_005115.4

NC_005114.4

NC_005112.4

NC_005111.4

NC_005110.4

NC_005108.4

NC_005107.4

NC_005106.4

NC_005105.4

NC_005104.4

NC_005103.4

NC_005102.4

NC_005101.4

NC_005100.4

JN235982.1

BK008127.1

NT_176400.1

NT_176130.1

NT_176064.1

XM_004999224.1

XM_004999332.1

XM_005005003.1

JN235984.1

AJ507121.1

NW_001581902.1

NW_001581969.1

NC_008803.1

NC_008805.1

EU024535.1

AJ233638.1

AJ233639.1

AJ233640.1

AJ233641.1

AJ233642.1

AJ233643.1

AJ507123.1

AJ507124.1

AJ233646.1

AJ233647.1

AJ233648.1

AJ233649.1

JN235986.1

J02063.1

JN235985.1

AJ233599.1

AJ233600.1

AJ233601.1

AJ233602.1

AJ233603.1

AJ233604.1

AF164896.1

AF164895.1

AF164898.1

HE680872.1

AF164888.1

AF164883.1

AF164906.1

AF164907.1

AF164900.1

AF164901.1

AF164902.1

DQ139724.1

DQ139725.1

DQ139726.1

DQ139727.1

DQ139728.1

DQ139729.1

DQ139730.1

DQ139731.1

DQ139732.1

DQ139767.1

DQ139768.1

DQ139769.1

DQ139770.1

DQ139771.1

DQ139772.1

DQ139773.1

AF317793.1

AF317794.1

AF317795.1

AF317796.1

AF309073.1

AF309074.1

AF312038.1

AF312039.1

AF312040.1

AF312041.1

AF312042.1

AF312043.1

AF312044.1

AF312045.1

AF312046.1

AF312047.1

AF312048.1

AF312049.1

AF312050.1

AF312051.1

AF312052.1

AF312053.1

AF312054.1

AF312055.1

AF312056.1

AF312057.1

AF312058.1

AF312059.1

AF312060.1

AF312061.1

AF312062.1

AF312063.1

AF312064.1

AF312065.1

AF312066.1

AF312067.1

AF312068.1

AF312069.1

AF312070.1

AF312071.1

AF312072.1

AF312073.1

AF312074.1

AF312075.1

AF312076.1

AF312077.1

AF312078.1

AF312079.1

AF312080.1

AF312081.1

AF312082.1

AF312083.1

AF312084.1

AF312085.1

AF312086.1

AF312087.1

AF312088.1

AF312089.1

AF312090.1

AF312091.1

AF312092.1

AF312093.1

AF312094.1

AF312095.1

AF312096.1

AF312097.1

AF312098.1

AF312099.1

AF312100.1

AF312101.1

AF312102.1

AF312103.1

AF312104.1

AF312105.1

AF312106.1

AF312107.1

AF312108.1

AF312109.1

AF312110.1

AF312111.1

AF312112.1

AF312113.1

AF312114.1

AF312115.1

AF312116.1

AF312117.1

AF312118.1

AF312119.1

AF312120.1

AF312121.1

AF312122.1

AF312123.1

AF312124.1

AF312125.1

AF312126.1

AF312127.1

AJ244593.1

AF151794.2

XM_003945265.1

XM_003944096.1

AJ233611.1

AJ233612.1

AJ233613.1

AY224371.1

AY224372.1

AJ233614.1

AJ233615.1

AJ233616.1

AJ233617.1

GQ906181.1

GQ906182.1

GQ906183.1

GQ906184.1

GQ906210.1

GQ906211.1

GQ906212.1

GQ906213.1

GQ906214.1

GQ906215.1

GQ906246.1

GQ906300.1

GQ906301.1

GQ906302.1

GQ906303.1

GQ906304.1

GQ906305.1

GQ906306.1

GQ906332.1

GQ906333.1

GQ906334.1

GQ906335.1

GQ906336.1

GQ906337.1

GQ906338.1

HQ456162.1

HQ456163.1

HQ456164.1

HQ456165.1

HQ456204.1

HQ456205.1

HQ456206.1

HQ456207.1

GQ906188.1

GQ906189.1

GQ906190.1

GQ906191.1

GQ906192.1

GQ906193.1

GQ906194.1

GQ906195.1

GQ906196.1

GQ906197.1

GQ906198.1

JN235983.1

DQ139747.1

DQ139757.1

DQ139758.1

DQ139759.1

DQ139760.1

DQ139761.1

DQ139762.1

DQ139763.1

DQ139764.1

DQ139765.1

DQ139766.1

KC010507.1

KC010519.1

JX406427.1

AY224358.1

AY224363.1

AY224364.1

AY224369.1

AY224370.1

AF164891.1

AF164908.1

AF164909.1

AF021929.1

AF164884.1

AF164905.1

K02914.1

AF164914.1

AF164915.1

AF164918.1

DQ247960.1

XM_003263508.1

NW_004087894.1

NC_019825.1

XM_003268373.2

XM_004089418.1

AB749815.1

AF164893.1

AF164910.1

AF164911.1

AY224330.1

AY224331.1

AY224376.1

AJ577598.1

AJ862653.1

AJ233657.1

AJ233658.1

AJ233659.1

AJ233660.1

AJ233596.1

AJ233597.1

AJ233598.1

AJ233672.1

AJ233605.1

AJ233606.1

AJ233607.1

AJ233608.1

AJ233609.1

AJ233610.1

GQ906185.1

GQ906186.1

GQ906187.1

GQ906216.1

GQ906247.1

GQ906248.1

GQ906249.1

GQ906307.1

GQ906308.1

GQ906309.1

GQ906339.1

GQ906340.1

GQ906341.1

GQ906342.1

HQ456166.1

HQ456167.1

HQ456168.1

HQ456208.1

KC010504.1

KC010516.1

AB749814.1

AY224329.1

AY224345.1

AY224346.1

AY224359.1

AY224360.1

KC010502.1

KC010514.1

AF164903.1

AF164904.1

AF164899.1

AY224377.1

GQ906164.1

GQ906165.1

GQ906166.1

GQ906167.1

GQ906224.1

GQ906225.1

GQ906226.1

GQ906227.1

GQ906228.1

GQ906260.1

GQ906261.1

GQ906262.1

GQ906263.1

GQ906264.1

GQ906265.1

GQ906280.1

GQ906281.1

GQ906282.1

HQ456144.1

HQ456145.1

HQ456146.1

HQ456147.1

HQ456177.1

HQ456178.1

HQ456179.1

HQ456180.1

HQ456181.1

KB102340.1

AJ233595.1

AJ233622.1

AJ233623.1

AJ233624.1

GQ906170.1

GQ906171.1

GQ906172.1

GQ906199.1

GQ906200.1

GQ906233.1

GQ906234.1

GQ906235.1

GQ906236.1

GQ906266.1

GQ906267.1

GQ906268.1

GQ906287.1

GQ906288.1

GQ906289.1

GQ906321.1

GQ906322.1

GQ906323.1

HQ456152.1

HQ456153.1

HQ456154.1

HQ456187.1

HQ456188.1

HQ456189.1

GQ906175.1

GQ906176.1

GQ906177.1

GQ906206.1

GQ906207.1

GQ906208.1

GQ906209.1

GQ906240.1

GQ906241.1

GQ906242.1

GQ906293.1

GQ906294.1

GQ906295.1

GQ906296.1

GQ906328.1

GQ906329.1

GQ906330.1

GQ906331.1

GQ906346.1

GQ906347.1

GQ906348.1

GQ906349.1

HQ456158.1

HQ456159.1

HQ456160.1

HQ456161.1

HQ456198.1

HQ456199.1

HQ456200.1

HQ456201.1

HQ456202.1

HQ456203.1

GQ906173.1

GQ906174.1

GQ906203.1

GQ906204.1

GQ906205.1

GQ906237.1

GQ906238.1

GQ906239.1

GQ906290.1

GQ906291.1

GQ906292.1

GQ906324.1

GQ906325.1

GQ906326.1

GQ906327.1

GQ906343.1

GQ906344.1

GQ906345.1

HQ456155.1

HQ456156.1

HQ456157.1

HQ456190.1

HQ456191.1

HQ456192.1

HQ456193.1

HQ456194.1

HQ456195.1

HQ456196.1

HQ456197.1

AB679508.1

AJ233618.1

AJ233619.1

AJ233620.1

AJ233621.1

GQ906159.1

GQ906160.1

GQ906217.1

GQ906218.1

GQ906219.1

GQ906220.1

GQ906250.1

GQ906251.1

GQ906252.1

GQ906253.1

GQ906254.1

GQ906255.1

GQ906272.1

GQ906273.1

GQ906274.1

GQ906275.1

GQ906310.1

GQ906311.1

GQ906312.1

HQ456136.1

HQ456137.1

HQ456138.1

HQ456139.1

HQ456169.1

HQ456170.1

HQ456171.1

HQ456172.1

GQ906161.1

GQ906162.1

GQ906163.1

GQ906221.1

GQ906222.1

GQ906223.1

GQ906256.1

GQ906257.1

GQ906258.1

GQ906259.1

GQ906276.1

GQ906277.1

GQ906278.1

GQ906279.1

GQ906313.1

GQ906314.1

GQ906315.1

GQ906316.1

HQ456140.1

HQ456141.1

HQ456142.1

HQ456143.1

HQ456173.1

HQ456174.1

HQ456175.1

HQ456176.1

GQ906178.1

GQ906179.1

GQ906180.1

GQ906243.1

GQ906244.1

GQ906245.1

GQ906297.1

GQ906298.1

GQ906299.1
